# *Metarhizium robertsii* ammonium permeases (MepC and Mep2) contribute to rhizoplane colonization and modulates the transfer of insect derived nitrogen to plants

**DOI:** 10.1371/journal.pone.0223718

**Published:** 2019-10-16

**Authors:** Soumya Moonjely, Xing Zhang, Weiguo Fang, Michael J. Bidochka

**Affiliations:** 1 Department of Biological Sciences, Brock University, St. Catharines, ON Canada; 2 Institute of Microbiology, Zhejiang University, Hangzhou, China; Chinese Academy of Agricultural Sciences Institute of Plant Protection, CHINA

## Abstract

The endophytic insect pathogenic fungi (EIPF) *Metarhizium* promotes plant growth through symbiotic association and the transfer of insect-derived nitrogen. However, little is known about the genes involved in this association and the transfer of nitrogen. In this study, we assessed the involvement of six *Metarhizium robertsii* genes in endophytic, rhizoplane and rhizospheric colonization with barley roots. Two ammonium permeases (*MepC* and *Mep2)* and a *urease*, were selected since homologous genes in arbuscular mycorrhizal fungi were reported to play a pivotal role in nitrogen mobilization during plant root colonization. Three other genes were selected on the basis on RNA-Seq data that showed high expression levels on bean roots, and these encoded a hydrophobin (Hyd3), a subtilisin-like serine protease (Pr1A) and a hypothetical protein. The root colonization assays revealed that the deletion of *urease*, *hydrophobin*, *subtilisin-like serine protease* and *hypothetical protein* genes had no impact on endophytic, rhizoplane and rhizospheric colonization at 10 or 20 days. However, the deletion of *MepC* resulted in significantly increased rhizoplane colonization at 10 days whereas Δ*Mep2* showed increased rhizoplane colonization at 20 days. In addition, the nitrogen transporter mutants also showed significantly higher ^15^N incorporation of insect derived nitrogen in barley leaves in the presence of nutrients. Insect pathogenesis assay revealed that disruption of *MepC*, *Mep2*, *urease* did not reduce virulence toward insects. The enhanced rhizoplane colonization of Δ*Mep2* and Δ*MepC* and insect derived nitrogen transfer to plant hosts suggests the role of *MepC* and *Mep2* in *Metarhizium*-plant symbiosis.

## Introduction

The endophytic insect pathogenic fungus (EIPF) *Metarhizium robertsii* (Clavicipitaceae) exhibits a varied lifestyle as an entomopathogen, endophyte or as a saprophyte [1]. *Metarhizium* spp. have been widely used as biocontrol agents against insect pests in agricultural fields. The molecular and biochemical factors regarding insect pathogenicity are relatively well studied [2,3]. Several *Metarhizium* spp. colonize the plant root and form a beneficial symbiotic relationship [1]. However, the genetic factors underpinning plant root or rhizospheric colonization in *M*. *robertsii* is largely unexplored.

*M*. *robertsii* forms a close symbiotic association with certain plants and is capable of transferring nitrogen from infected insects [4,5] to the plant host in exchange for carbon [6]. Relative to the knowledge on the genetic attributes of insect pathogenesis, little is known about the complex relationship between *Metarhizium* and plant roots. The importance of *Metarhizium* raffinose transporter (*Mrt)* [7] and *Metarhizium* invertase (*MrINV*) [8] during interaction with plants has been demonstrated. Most plants rely on nitrogen fixing microorganisms or microbial decomposers to acquire nitrogen from the soil. The involvement of nitrogen transporters, ammonium permease and urease, are reported in arbuscular mycorrhizal (AM) fungi during plant colonization [9–13]. The two main nitrogen transporters involved in the nitrogen regulatory network in fungi are ammonium permeases and amino acid permeases [14]. AM fungi acquire nitrogen from the soil in the form of ammonium, nitrate or amino acids, which are then transported to host plants through ammonium/methylammonium permease (AMT/Mep) transporters. Here inorganic nitrogen or ammonia is assimilated by AM fungi and subsequently converted to arginine in the extraradical mycelium by nitrate reductase or glutamine synthase/glutamate synthase. Arginine is then translocated to the intraradical mycelium where it is converted to urea and ornithine. Urea is then converted to NH_4_^+^ by the action of urease, which is subsequently mobilized to the plant via ammonium transporters/permeases [13]. The symbiotic interaction of *Metarhizium* with plants [6] suggests the involvement of fungal nitrogen transporters. Nevertheless, genes involved in nitrogen transfer in *M*. *robertsii* during plant symbiosis are unknown. Furthermore, *Metarhizium* share similarities with mycorrhizal fungi in terms of forming symbiotic association and facilitating nutrient exchange with plants, hence it is hypothesized that similar nitrogen transfer mechanisms may be operating in *Metarhizium* during symbiotic association with plants.

*Metarhizium* expresses different subsets of genes as a means of physiological adaptation under various conditions. *Metarhizium* spp. demonstrated large scale differences in gene expression patterns during growth in plant root exudate, insect cuticle or hemolymph as well as in insect host specialization [15–18]. Although the symbiotic ability and the role as a plant growth promoter has been demonstrated, little is known about the genes involved in this symbiosis. In order to get a better understanding of the genes involved during the symbiotic association of *Metarhizium* with plants, RNA-Seq was performed on *Metarhizium* transcripts during bean root colonization and three genes that showed high expression levels were selected for further analysis. A genome survey of *M*. *robertsii* revealed the presence of two ammonium permeases (*MepC* and *Mep2*) and a urease gene, which has also been selected for the study. Over all, we investigated the involvement of 6 different genes *ammonium permease C* (*MepC*; Gene number—*MAA_04182)*, *ammonium permease C* (*Mep2;* Gene number—*MAA_05002*), *urease* (Gene number—*MAA_07458)*, *subtilisin-like serine protease deletion Pr1A* (Gene number—*MAA_05675*), *hypothetical protein* (Gene number—*MAA_08959*), *hydrophobin 3* (Gene number—*MAA_10298)* in *M*. *robertsii* in barley root colonization and insect pathogenesis by using targeted gene deletions. *M*. *robertsii* adhesin 2 deletion (Δ*Mad2*) and a raffinose transporter deletion (Δ*Mrt)* strains were used as comparisons for the plant root colonization assays. In addition, we also quantified the ability of Δ*MepC*, Δ*Mep2* and Δ*Urease* mutants to transfer insect derived nitrogen to barley plants.

## Materials and methods

### Fungal strains and culture conditions

*Metarhizium robertsii* (ARSEF 2575) wild-type (WT) strain (U.S. Department of Agricultural Research Service Collection of Entomopathogenic Fungal Cultures, Ithaca, NY) was grown and maintained on Potato Dextrose Agar (PDA) (Bioshop Inc.) at 27°C. *M*. *robertsii ammonium permease C* deletion (*ΔMepC*), *ammonium permease 2* deletion (*ΔMep2*), *ΔUrease*, *subtilisin-like serine protease* deletion (*ΔPr1A*), *ΔHypothetical protein (*Δ*Hypo*. *protein)* and *hydrophobin* 3 deletion (*ΔHyd3*) strains were used in this study. *M*. *robertsii* adhesin 2 deletion (Δ*Mad2*) [19] and a *Metarhizium* raffinose transporter deletion (Δ*Mrt*) [7] strains were used as comparisons for the plant root colonization assays and were kindly provided by Dr. R.J. St. Leger (University of Maryland). The fungal cultures were routinely grown and maintained on PDA as needed. Conidia were obtained from 12–14 day old PDA plates and conidia were recovered with 0.01% Triton X-100. Conidial yields were then quantified using a hemocytometer and the concentration was adjusted to 1x10^7^ conidia/ml.

### Targeted gene deletions

Gene deletions based on homologous recombination was conducted according to our previously developed high-throughput methodology [20]. The plasmids for gene deletion were constructed using Gateway Bp Clonase II Enzyme Mix (Invitrogen) or restriction enzyme digestion and ligation. The primers for deletion plasmid construction and other primers used in this study are presented in S1 Table. All PCR products were cloned using high-fidelity Taq DNA polymerase (KOD) and confirmed by DNA sequencing. PCR was performed to verify the targeted gene deletions (S1 Fig).

### Growth rate, conidial germination and stress sensitivity assay

The radial growth of *M*. *robertsii* WT and gene deletion strains was examined by inoculating the center of the PDA plate with 10 μl of the conidial suspension (1x10^7^ conidia/ml), incubated at 27°C and colony diameter was measured on day 3, 7 and 14. For the stress sensitivity assay, 10 μl of the conidial suspension (1x10^7^ conidia/ml) was inoculated on PDA plates supplemented with either 0.01% SDS or 100 μg/ml Congo red. The plates were incubated at 27°C in the dark and the colony diameters were recorded after 7 days [21]. All assays were performed with 5 biological replicates and the assays were repeated with independent batches of conidial suspension.

The hydrophobicity of Δ*Hyd3* was assessed using a wettability test [22]. The test was performed by placing 10 μl of a water droplet on the surface of a 14 day old culture and the contact angle of the water droplet was observed after 10 minutes, 30 minutes and 1 hour.

### RNA-Sequencing of *Metarhizium* colonized plant root

Briefly, *M*. *robertsii* were allowed to colonize seven day old germinated bean seedlings (*Glycine max*, Ontario Seed Co Ltd., Ontario, Canada) in sterile soil under greenhouse conditions (25°C with a photoperiod of 16:8 h light: dark cycle with relative humidity maintained between 60–80%) for 7 days; thereafter the root was harvested, washed, macerated and RNA was extracted using QIAzol lysis reagent (Qiagen) following manufacturer’s protocol. Total RNA was extracted from *Metarhizium* colonized bean roots using QIAzol (Qiagen), following manufacturer’s instructions and the concentration of each RNA samples was quantified using Qubit fluorometer (Invitrogen).The purity and the integrity of the RNA samples were assessed spectrophotometrically and agarose gel electrophoresis respectively. The reverse transcription of the sample was prepared and sequenced with the SOLiD^TM^ 4 (Life Technologies) system. One replicate of the *Metarhizium* colonized bean root was used for RNA-Seq analysis. *Metarhizium* transcripts were identified and differentiated from plant transcripts by transcript comparison to the *Metarhizium* genome as the template.

Real-time PCR validation of five *Metarhizium* genes was conducted to confirm the RNA-Seq results. The primer sequences used for real-time PCR validation are given in S1 Table. Fungal conidial suspension (1x10^6^ conidia/ml) was inoculated in potato dextrose broth (PDB) and cultures were incubated at room temperature (22°C) at 120 r.p.m. Fungal mycelia was collected after 4 days by vacuum filtration and ~0.25 g of fungal mycelia was transferred to flasks containing 25 ml of bean root exudate (BRE) and potato dextrose broth (PDB). The haricot bean (*Phaseolus vulgaris*, Ontario Seed Co Ltd., Ontario, Canada) root exudate was prepared as described [23]. Cultures were then incubated at room temperature (22°C) at 120 r.p.m and the fungal mycelia was then harvested from bean root exudate after 8 hours of incubation. Total RNA was extracted from fungal mycelia using QIAzol (Qiagen), following manufacturer’s instructions. The reverse transcription reactions of each sample were generated using the high capacity cDNA reverse transcription kit (Applied Biosystems) with 4 μg of total RNA in total volume of 80 μl, following manufacturer’s recommendations. The transcript levels of each genes were assessed via real-time PCR (BioRad CFX96 real-time PCR system) using SensiFast^™^ SYBR No-ROX kit (Bioline). The real-time PCR reaction mix contained 2 μl of cDNA, 0.4 μl of 10 μM forward and reverse primers, 5 μl of SensiFast^™^ SYBR No-ROX mix and 2.2 μl of nuclease free water in a final volume of 10 μl. The real-time PCR was performed under following conditions: 95°C (2 mins), 40 cycles of 95°C (5 secs), 62–64°C (10 secs) and 72°C (20 secs). The specificity of the PCR products was verified by agarose gel electrophoresis. BioRad CFX Maestro (Version: 4.1) was used to analyze the expression of the selected genes using glyceraldehyde-3-phosphate dehydrogenase (gpd) as reference gene. The real-time PCR was performed using three biological replicates and three technical replicates were used for each biological replicate.

### Root colonization assay

Root colonization assays were performed using barley (*Hordeum vulgare*, Sprout Master, Ontario, Canada) as the host plant. The endophytic, rhizoplane and rhizospheric association of WT and gene deletion strains were analyzed. Seeds were surface sterilized with three washes in 4% sodium hypochlorite (NaOCl) for 5 minutes each. The seeds were rinsed with sterile distilled water after each NaOCl wash. The seeds were kept overnight at 4°C for synchronization of growth before planting. The seeds were then allowed to germinate in water agar (1%) for 3–4 days at 25°C. The germinated seedlings were then planted in sterile vermiculite (Ther-O-Rock East Inc.). Fungal inoculations were performed using the drench method [24] where 5 ml of the conidial suspension was poured onto the vermiculite surface of each pot. The plants were kept in a greenhouse at 25°C with a photoperiod of 16:8 h light: dark cycle with relative humidity maintained between 60–80%. The plants were watered daily with sterile distilled water. Five biological replicates were prepared for each treatment. To quantify fungal association, barley roots were harvested from 10 and 20 day old plants. The amount of fungal association on barley roots was analyzed as described previously [23]. To examine the endophytic association, the harvested roots were first washed in water to remove the attached vermiculite. The washed roots were then immersed in 2% NaOCl for 10 seconds and finally rinsed with sterile distilled water to remove traces of NaOCl. The roots were then cut into ~2-5mm pieces, weighed and homogenized (Biospec products Inc.) in sterile distilled water for 2 mins. The homogenized root samples were then plated on modified CTC agar (PDA supplemented with 0.5 g/l chloramphenicol, 0.004 g/l thiabendazole and 0.5 g/l cycloheximide) [25] and colony forming units (CFU) values were calculated as CFU/g of root weight. To check the rhizoplane colonization, the harvested roots were treated as described above except the 2% NaOCl treatment was omitted. Water inoculated barley plants were used as controls. Rhizospheric populations of WT and gene deletion strains were also monitored. Here, the vermiculite attached to the barley roots was collected during the harvest, weighed and suspended in 0.01% Triton X-100. The serial dilutions of the suspension were plated on CTC media and the CFU were calculated for per gram of vermiculite.

### Insect bioassays

The virulence of the WT *M*. *robertsii* and gene deletion strains were assayed against larvae of *Tenebrio molitor* and *Galleria mellonella*. An aliquot of 10 μl of the conidial suspension (1x10^7^ conidia/ml) was applied to the cuticle of the larvae. Each larva was placed separately in 60 mm x 15 mm diameter Petri dish. Humidity was maintained in each Petri dish with a moistened filter paper. The treated larvae were housed at 25°C and daily mortality rate was recorded. Each replicate contained 20 larvae and the experiment was duplicated. Controls were 0.01% Triton X-00 treated larvae. The LT_50_ values were calculated using Probit analysis.

### ^15^N-labelled nitrogen transfer assay

The ability of Δ*MepC*, Δ*Mep2* and Δ*Urease* to translocate nitrogen from ^15^N -labeled wax moth larvae to the leaves of barley was quantified by the microcosm method as previously described [4,5]. A 10 μl solution of ^15^N-labelled ammonium sulphate (5%) was injected through the rear proleg of wax-moth larvae. After 48 hours the larvae were infected with 14 day old fungal conidia. The infected larvae were then placed into microcosms containing autoclaved vermiculite as previously described [4]. The microcosm was then placed in the pots and covered with autoclaved vermiculite and the 3 day old germinated barley seedling was planted on each pot. The ^15^N transfer was assessed by the nitrogen transporter mutant strains using two treatments. In the first experimental set, plants were watered daily with sterile distilled water and once a week with 25 ml of 50% Modified Melin-Norkrans (MMN) solution (0.05 g CaCl_2_, 0.025 g NaCl, 0.05 g KH_2_PO_4_, 0.5 g (NH4)_2_PO_4_, 0.15 g MgSO_4_·7H_2_O, 1 mg FeCl_3_·6H_2_O,5g glucose monohydrate, 10 ml trace element solution [3.728 g KCl, 1.546 g H_3_BO_3_, 0.845 g MnSO_4_·H2O, 0.05 g ZnSO_4_·7H_2_O, 0.0125 g CuSO_4_, 0.05 g (NH_4_)_6_Mo_7_O_24_·4H_2_O per 1 liter] per 1 liter). In the second treatment, plants were watered daily with sterile distilled water but 50% MMN solution was omitted. The amount of ^15^N transfer to plant tissues was determined by harvesting the above ground plant parts after 10 and 20 days. The harvested plant material was dried at 60°C for 24 hours and were then crushed into a fine powder using a mortar and pestle. The ground plant material was encapsulated in 4-mm by 4-mm tin cups and analyzed for ^15^N content by using an NOI-5 emission spectrophotometer.

### Nitrogen source assay

The ability of *ΔMepC*, *ΔMep2*, *ΔUrease* and WT to grow on different nitrogen sources was assessed on basal salt agar (BS; 0.1% KH_2_PO_4_, 0.025% Na_2_SO_4_, 0.05% KCl, 0.0125% MgSO_4_·7H_2_O, 0.00625% CaCl_2_, and 1% glucose) [7] supplemented with different nitrogen sources (1 or 30 mM (NH_4_)_2_ SO_4_, 30 mM arginine, 30 mM glutamine, 30 mM glutamate, 1 or 30 mM proline or 30 mM urea). A 1x10^7^ conidial/ml suspension (5 *μ*l) was spot inoculated onto BS agar supplemented with different nitrogen sources and growth rates were compared with the WT after 7 days.

Ammonium production, based on pH of the gene deletion strains and WT, was also examined. Here, 1 ml of the conidial suspension was added on to potato dextrose broth and allowed to grow for 4 days at 100 rpm at 27°C. Mycelia was then filtered, washed with sterile distilled water and transferred to minimal media (0.02% KH_2_PO_4_, 0.01% MgSO_4_, 0.2ppm FeSO_4_, 1 ppm ZnSO_4_, 0.02 ppm CuSO_4_, 0.02 ppm MnMoO_4_, 0.02 ppm MnCl_2_) supplemented with different amino acids (10 mM glutamate, glutamine or arginine). The fungal mycelia (2.5 g) was added to 100 ml of minimal media broth and incubated at 100 rpm at 27°C. The samples were collected at 0, 6, 24 and 48 hours and the pH were recorded.

### Phylogenetic analysis

The amino acid sequences analysis of ammonium permeases of *M*. *robertsii* and other fungal ammonium transporters was conducted. Sequence alignments were generated using MUSCLE v3.7 with default parameters [26], and these were then manually refined and end-trimmed to eliminate poor alignments and divergent regions. Unambiguously aligned positions were used for constructing phylogenetic trees with Maximum Likelihood (ML), Bayesian Inference (BI) or distance-based Neighbor-Joining (NJ). A ML tree was constructed using MEGA6.0 (gap treatment: use all sites; 100 bootstrap replications) [27]. The optimal model of phylogenetic relationship was determined using the Find Best Protein Model provided by MEGA 6.0 [27]. A Bayesian inference tree was constructed with MrBayes v3.2.5 as described [28]. The best model was determined as above using MEGA 6.0. For each BI analysis, we used four Metropolis-coupled chains and ran them for 5,000,000 generations, sampling every 1000 generations (‘mcmc ngen = 5000000 sample frequency = 1000’). The analysis finished with an average standard deviation of split frequencies of 0.01 or less. The first 25% of trees were discarded as “burn-in”. A NJ tree was constructed with default parameters (gap treatment: pairwise deletion; 1000 bootstrap replications) using NJ in MEGA6.0 [27]. Bootstrap support values were obtained by generating 1,000 pseudo-replicates.

## Results

### RNA-Sequencing and transcriptome analysis

RNA-Sequencing (RNA-Seq) of *Metarhizium* transcripts during bean root association revealed the expression of over 4000 *Metarhizium* genes. In the transcriptome data, 217 genes showed relatively higher expression (10–234 readings) and 25% of these upregulated genes were putative uncharacterized or hypothetical protein. Of these, the top five most highly expressed *M*. *robertsii* genes (~90–234 readings) were hydrophobin (*MAA_10298*), tubulin beta chain (*MAA_02081*), subtilisin-like serine protease *Pr1A* (*MAA_05675*) and two other genes that encode putative uncharacterized proteins (*MAA_08959* and *MAA_00771*). The protein sequence of *MAA_08959* (hypothetical protein) was surveyed in GenBank, and similar sequences were reported in other *Metarhizium* species, however these were also categorized as putative uncharacterized or hypothetical protein. A homolog of *MAA_08959* was reported in *M*. *brunneum* as filamin/ABP280 repeat like protein but was not functionally characterized. S2 Table shows the top 10 *M*. *robertsii* genes that showed increased expression in bean root (RNA-Seq of <1% of total transcripts).

The transcript levels of *Hypo*. *protein* (*MAA_08959*), *Hyd3* (*MAA_10298*), *Pr1A* (MAA_05675), *tubulin beta chain* (*MAA_02081*), and *hydrophobin-like protein ssgA* (*MAA_09731*) were verified by conducting real-time reverse transcriptase PCR analysis from fungal RNA grown in BRE and PDB. All transcripts except *tubulin beta chain* showed significantly (p<0.001) higher transcript levels (S2 Fig) in BRE compared to PDB.

### Phylogenetic analysis

**Fig 1:**
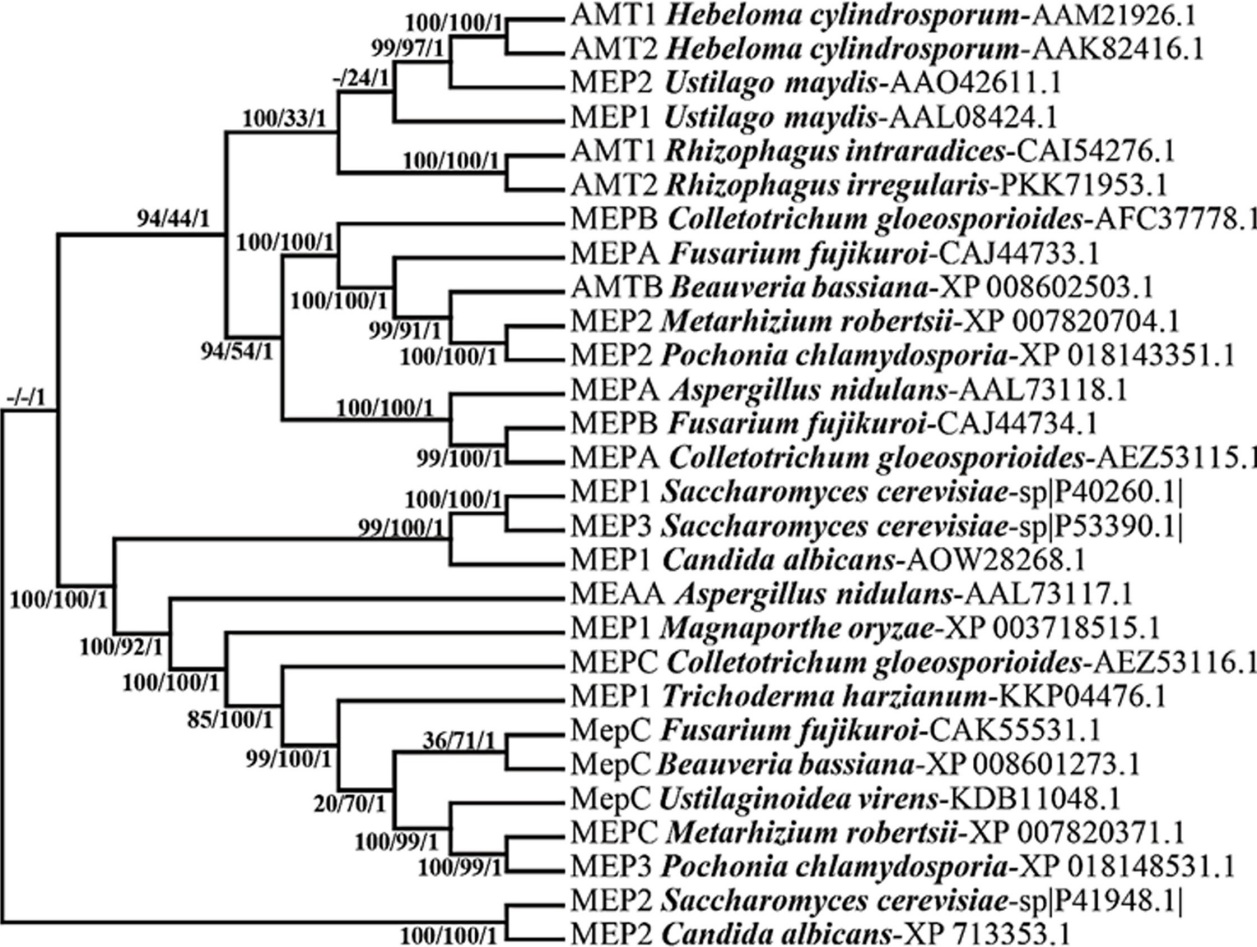
Phylogenetic relationship between the amino acid sequences of ammonium permeases of *M*. *robertsii* and other fungal ammonium transporters. The phylogenetic tree was created using Maximum Likelihood (ML), Bayesian Inference (BI) and Neighbor joining (NJ). The Mep2 of *Saccharomyces cerevisiae* sequence was used to root the tree. The sequences of other fungal transporters were obtained from GenBank database with the following accession numbers: *Aspergillus nidulans* (AnMEAA: AAL73117,AnMEPA: AAL73118), *Beauveria bassiana* (MEP3: PMB65720.1), *Candida albicans* (MEP1: AOW28268.1, MEP2: XP 713353.1), *Colletotrichum gloeosporioides* (MEPA: AEZ53115.1, MEPB: AFC37778.1, MEPC:AEZ53116.1), *Fusarium fujikuroi* (FfMEPA: CAJ44733,FfMEPB: CAJ44734, FbMEPC: CAK55531), *Glomus intraradices* (GintAMT1: CAI54276; GintAMT2:CAX32490), *Hebeloma cylindrosporum* (HcAMT1: AAM21926, HcAMT2: AAK82416, HcAMT3: AAK82417), *Metarhizium robertsii* (MepC: XP_007820371.1, Mep2:XP007820704.1), *Rhizophagus irregularis* (AMT2: PKK71953.1), *Rhizophagus intraradices* (AMT1: CAI54276.1), *Saccharomyces cerevisiae* (ScMEP1: P40260, ScMEP2: P41948, ScMEP3: P53390), *Trichoderma harzianum* (MEP1 KKP04476.1), *Ustilago maydis* (UmMEP1: AAL08424, UmMEP2: AAO42611), *Ustilago virens* (MepC; KDB11048.1). The values adjacent to each internal node represents the bootstrap support values.

BLAST search using the NCBI protein database of MepC showed amino acid sequence similarities to the ammonium transporters of phytopathogens and other insect pathogens/plant root colonizers, with more than 80% similarity to other Hypocreales such as *Ustilaginoidea virens*, *Beauveria bassiana* and *Trichoderma harzianum* (Fig 1). These fungi represent diverse lifestyles as phytopathogens, endophytic insect pathogens and endophytic mycoparasites, respectively. Phylogenetic analysis showed that Mep2 amino acid sequences clustered with ammonium permeases from phytopathogenic and endophytic fungi. Mep2 showed a close phylogenetic relationship with ecto/endo mycorrhizal ammonium transporters, while MepC formed a distant clade with mycorrhizal ammonium transporters.

The BLAST analysis of urease showed more than 80% amino acid sequence similarities with *Purpureocillium lilacinum* (entomopathogenic, rhizospheric), *Tolypocladium capitatum* (insect parasitic, mycoparasitic), *Hirsutella minnesotensis* (insect and nematode pathogenic) and other endophytic/phytopathogenic fungi (*Trichoderma* spp., *Colletrotrichum* spp., *Verticillium* spp. and *Fusarium* spp.). The nucleic acid sequence analysis of *MAA_10298* revealed sequence identity with a functionally characterized Class I hydrophobin from *M*. *brunneum*, HYD3, and hence named as *Hyd3* in this study.

### Effect of gene disruption on phenotypic characteristics

**Fig 2:**
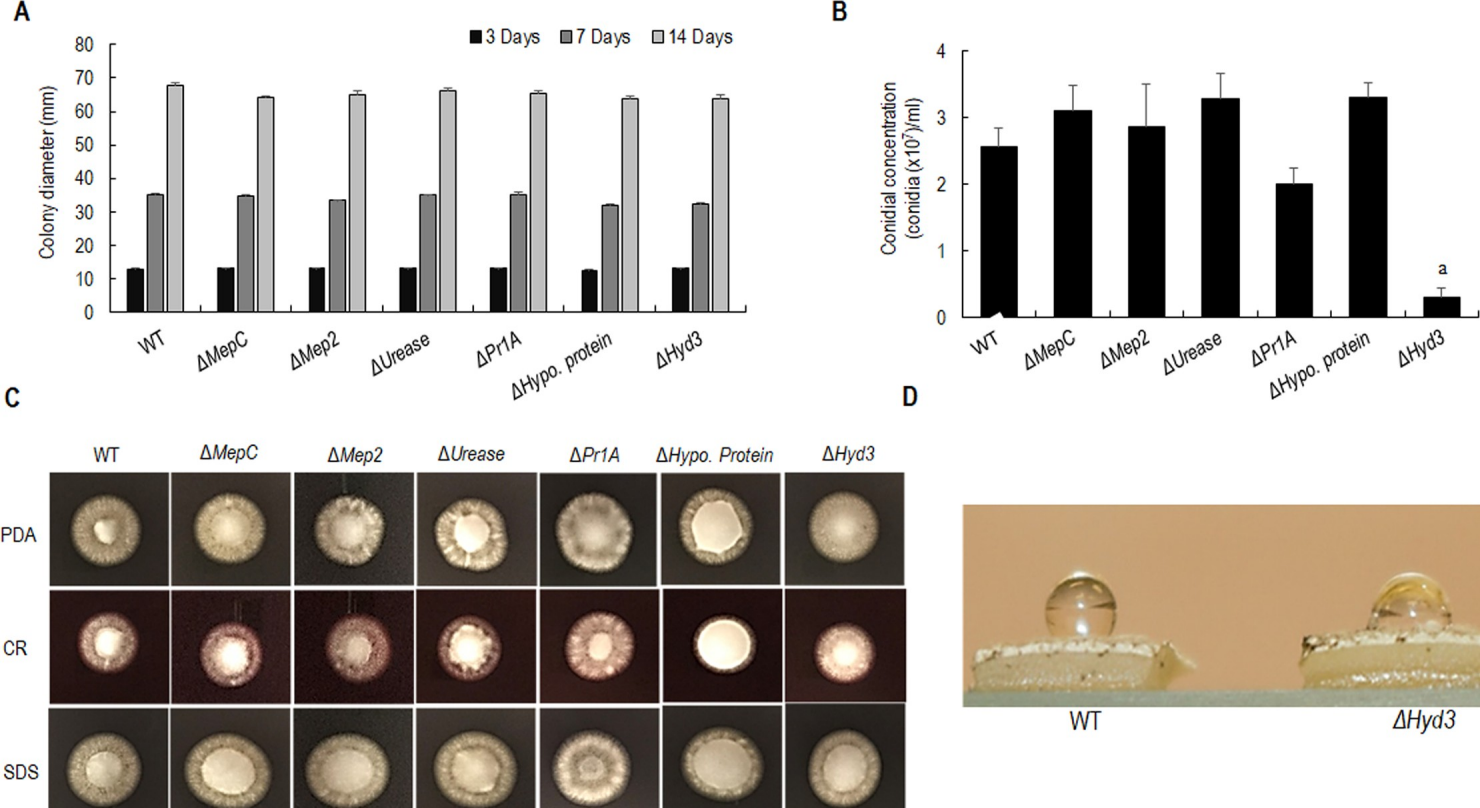
**(A) Growth rate of *M*. *robertsii* and mutant strains on potato dextrose agar medium**. 10μl of the 1x10^6^ conidia/ml was spot inoculated on solid agar medium and incubated at 27°C. The colony diameter was measured on 3, 7 and 14 days. The error bars represent the standard error based on 5 biological replicates. **(B) Quantification of conidial production of *M*. *robertsii* and mutant strains.** Different letters indicate statistically significant differences relative to WT, ‘a’ indicates p<0.001. (**C**) Colony morphology of WT and mutants in PDA and response stress conditions, 0.01% SDS, 100 μg/ml Congo red (CR). (**D) Water droplet hydrophobicity test of WT and *Hyd3* mutant.** Image shown was taken after 10 mins. The difference in the contact angle of the water droplet is wide for *Hyd3* mutant compared to WT.

The growth rate of WT *M*. *robertsii* and the gene deletion strains were similar on PDA at 27°C (Fig 2A). Disruption of *Hyd3* affected conidiation on PDA and conidial yield was decreased by ~7 fold compared with WT (ANOVA, p<0.001) (Fig 2B). No significant differences in conidiation were noted for other gene deletion strains relative to WT (Fig 2B). However, differences in the colony morphology were noted in gene deletion strains relative to WT. The Δ*Hypo*. *protein* showed a thick white cottony mycelium (Fig 2C) compared to WT on PDA. The effect of various stress conditions on the growth of gene deletion strains was assayed on agar plates containing either Congo red or SDS. No significant differences in the growth rate were observed in any of the stress conditions (Fig 2C). The hydrophobicity test showed that Δ*Hyd3* was a wettable phenotype observed as a decrease in the contact angle of the water droplet on the colony surface (Fig 2D). The WT retained the water droplet after 1 hour while Δ*Hyd3* exhibited a less hydrophobic colony surface.

### Effect of gene disruption on insect pathogenesis

**Fig 3:**
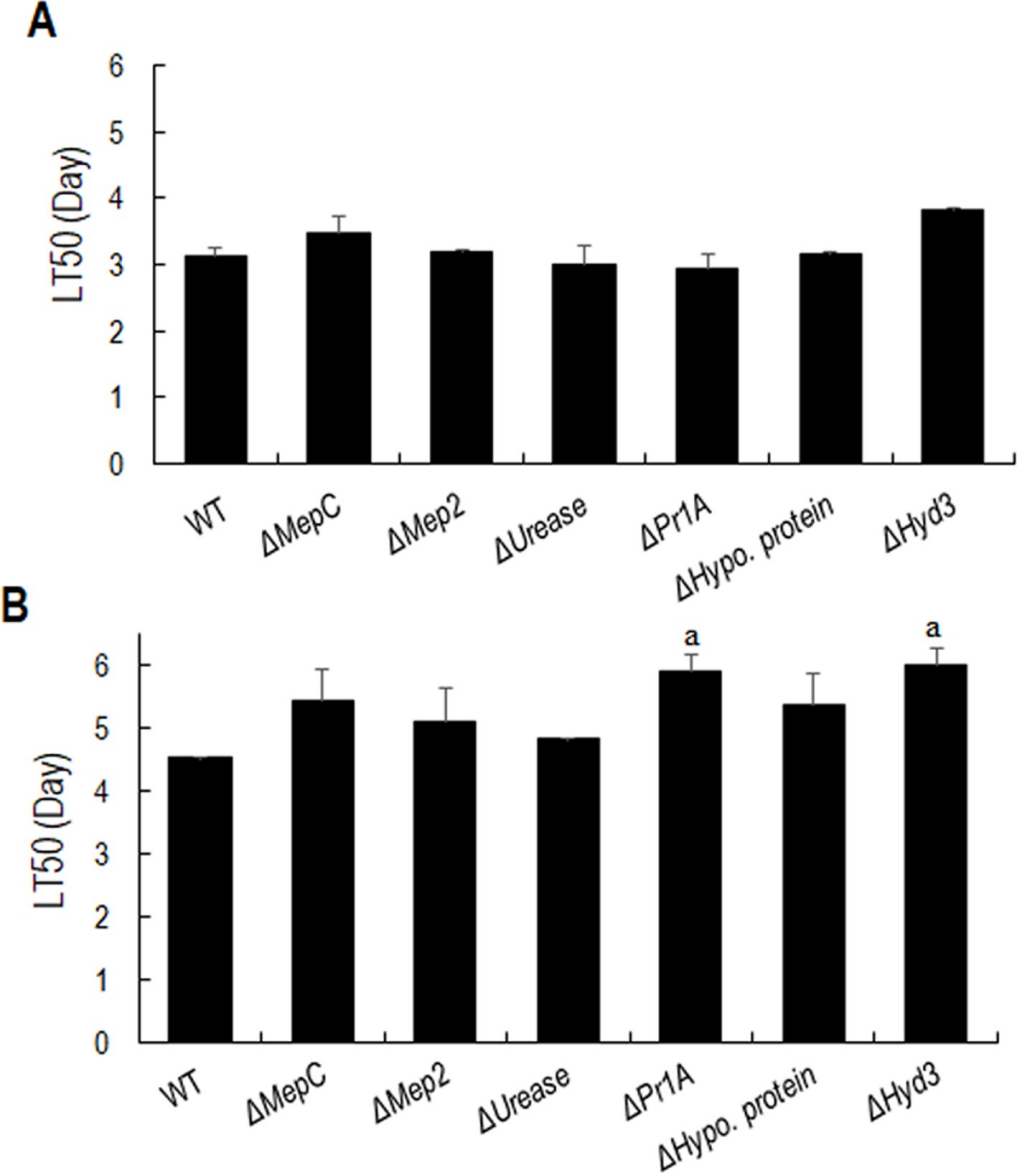
Insect bioassays. The calculated LT_50_ values for *M*. *robertsii* WT and mutants on topical application to **(A)** *Galleria mellonella*, **(B)** *Tenebrio molitor*. The error bar represents the standard deviation of two replicates and each replicate contained 20 larvae. Letters indicate statistically significant differences from WT, ‘a’ indicates p<0.05.

Pathogenicity assays against *Galleria mellonella* larvae revealed no significant reduction in the mortality by the gene deletion strains (Fig 3A). Similar results were obtained for *Tenebrio molitor* larvae bioassays except that *ΔPr1A* (LT_50_ 5.90±0.28) and *ΔHyd3* (LT_50_ 6.02±0.56) showed reduced virulence (p = 0.04) relative to the WT (LT_50_ 4.53±0.1) (Fig 3B).

### Effect of gene disruption on rhizoplane and endophytic colonization ability

**Fig 4:**
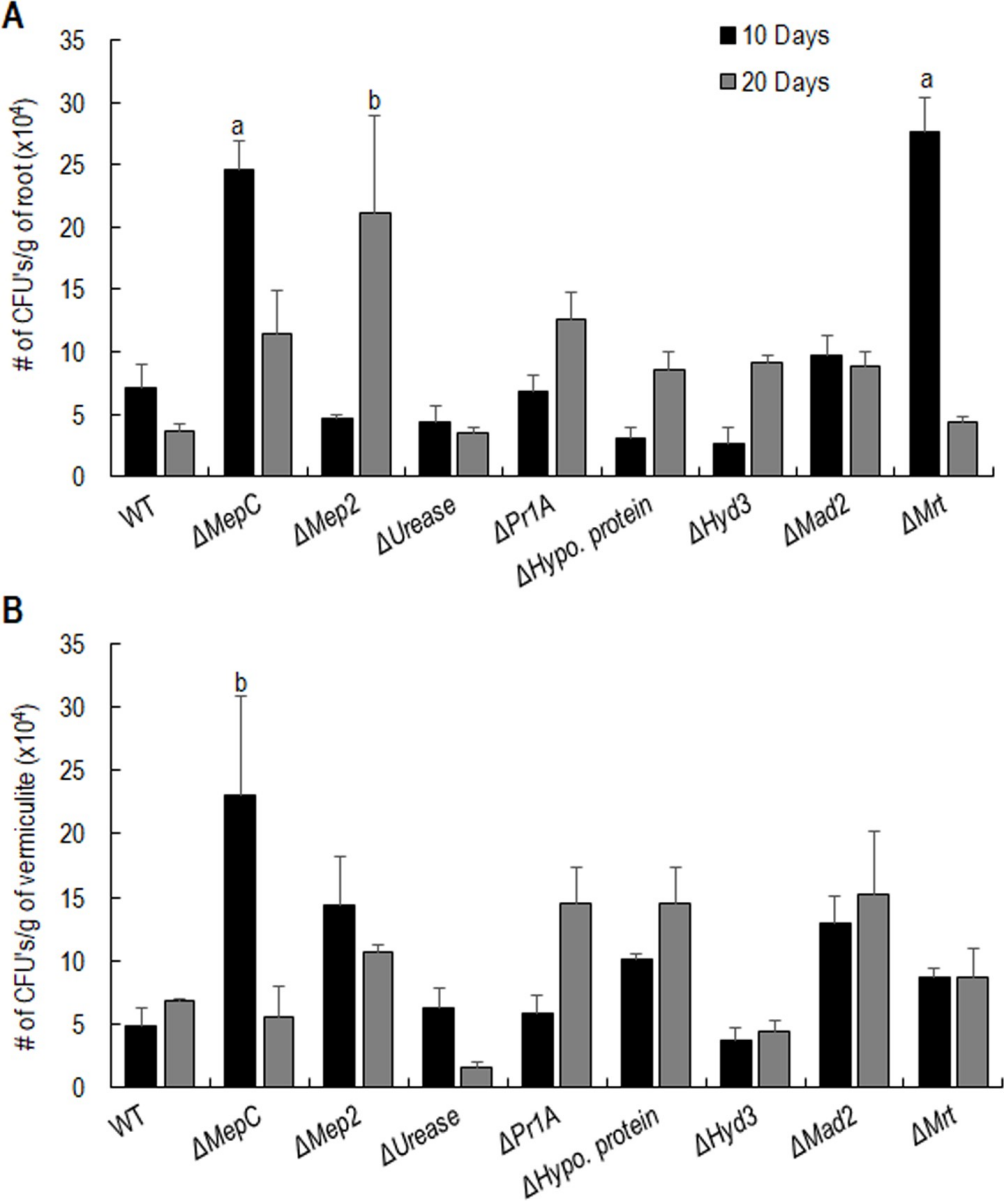
Rhizoplane and rhizosphere colonization of *M*. *robertsii* WT and mutant strains. **(A) Rhizoplane colonization.** 3–4 days old germinated barley seedlings were planted on sterile vermiculite inoculated with 5 ml of 10^7^ conidia/ml. Barley roots were harvested on 10 and 20 days post inoculation and washed in water. The roots were then homogenized and plated on selective media plates. The CFUs were counted after 7 days and CFUs/g of root weight was calculated. **(B) Rhizosphere colonization.** ~1±0.25 g of vermiculite surrounding root was collected on the day of 10 and 20 day harvest. Vermiculite was suspended in 0.01% Triton X-100 and 0.1 ml was plated on selective media after serial dilution. The CFU’s were counted after 7 days and CFUs/g of soil weight was calculated. The error bars represent the standard error for 5 biological replicates and different letters indicate statistically significant differences relative to WT. ‘a’, p<0.008; ‘b’, p<0.02.

The ammonium permease deletions, Δ*MepC* and Δ*Mep2* showed increased rhizoplane colonization after 10 or 20 days respectively compared to the WT (Fig 4A). A significant increase in CFU was observed for Δ*MepC* (24.5x10^4^ CFU/g of root) at 10 days post treatment as compared to WT (7.1x10^4^ CFU/g of root) (Tukeys multiple comparison test: WT v/s Δ*MepC* (p<0.001)). However, Δ*MepC* showed no significant difference in rhizoplane colonization levels relative to WT after 20 days. In contrast, Δ*Mep2* showed similar levels of root colonization as the WT after 10 days; however, significantly higher CFU levels (35.5 x10^4^ CFU/g of root) was recovered after 20 days (WT v/s Δ*Mep2* (p<0.0001). The sugar transporter deletion Δ*Mrt* showed a similar rhizoplane colonization pattern as Δ*MepC*. Rhizoplane colonization for Δ*Mrt* (27.7x10^4^ CFU/g of root) after 10 days post treatment was ca. 4 times greater than WT. Nevertheless, the recovery of Δ*Mrt* (4.3x10^4^ CFU/g of root) from plant roots after 20 days post inoculation was similar to the WT (3.6x10^4^ CFU/g of root). The disruption of the *urease* gene in *M*. *robertsii* had little impact on root colonization. Similarly, the disruption of *Hyd3*, *Pr1A* and *Hypo*. *protein* did not affect the rhizospheric or rhizoplane colonization abilities compared with the WT. When the rhizospheric persistence of these strains in vermiculite was analyzed, there were no significant differences in the CFU recovered from vermiculite compared to WT except for Δ*MepC* at day 10 (Fig 4B). No significant differences in endophytic colonization were observed for the gene deletion strains compared with the WT (S3 Table).

### Insect derived ^15^N transfer to barley by *Metarhizium* nitrogen transporter mutants

**Fig 5:**
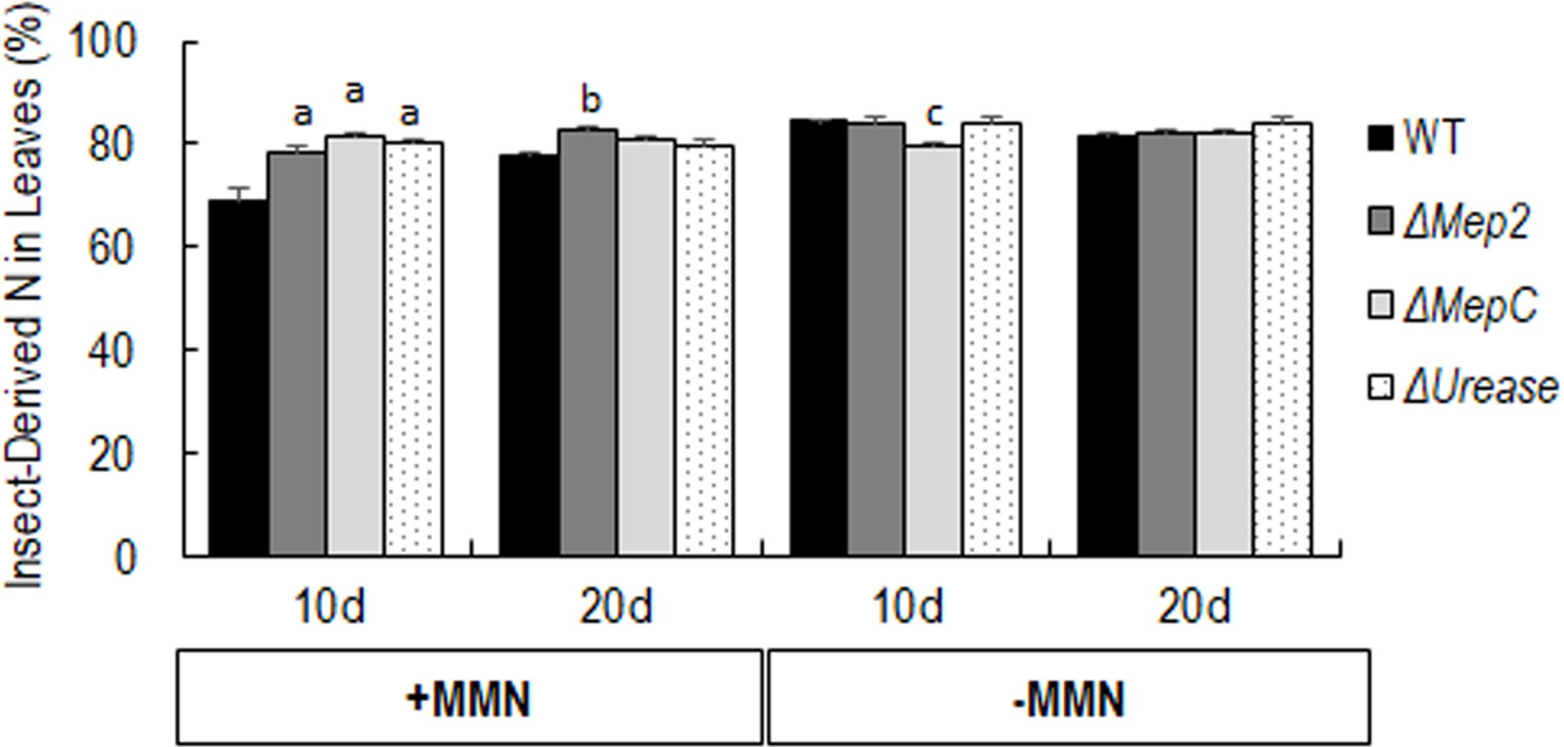
Percentage of plant nitrogen derived from ^15^N-injected wax moth larvae by WT *Metarhizium*, Δ*Mep2*, Δ*MepC* and Δ*Urease*. Two conditions were used: Plants treated with 25 ml of 50% MMN (+MMN) and without 50% MMN (-MMN). Amount of insect-derived nitrogen in barley leaves were determined by NOI-5 emission spectrophotometer after 10 (10d) and 20 (20d) days. The error bars represent the standard error for 6 biological replicates and the letters indicate statistically significant differences relative to WT. ‘a’, p <0.0001; ‘b’, p<0.005; ‘c’, p<0.03.

*Metarhizium* strains including WT and the nitrogen transporter mutants were able to transfer significant amounts of insect derived-^15^N to barley after 10 and 20 days of growth (Fig 5). *M*. *robertsii* WT showed significant difference in the insect derived-^15^N incorporation between MMN treated and untreated plants after 10 days of growth in microcosms. A significantly lower ^15^N incorporation was noted in MMN treated plants (68.84%) versus untreated plants (84.33%) when grown in microcosms containing *M*. *robertsii* WT infected, ^15^N injected wax moth larvae. However, no significant difference was observed in insect derived-^15^N incorporation between MMN treated and untreated after 20 days of growth in the presence of WT infected, ^15^N-injected wax moth larvae.

All three of the nitrogen transporter mutants showed significantly higher ^15^N incorporation compared to WT in MMN treated barley plants after 10 days of growth in the presence of microcosms containing ^15^N-injected wax moth larvae. After 10 days of growth, Δ*MepC* (81.25%) showed significantly higher insect derived-^15^N transfer to barley leaves relative to the WT (68.84%) in MMN treated plants. In contrast, plants that were not treated with MMN, Δ*MepC* (79.75%) showed significantly less ^15^N transfer compared to WT (84.33%) (Tukeys multiple comparison test: WT v/s Δ*MepC* (p<0.03)). However, no significant difference in insect derived-^15^N incorporation by Δ*MepC* was observed relative to WT in both MMN treated and untreated plants after 20 days of growth.

In the MMN treated barley plants, Δ*Mep2* showed significantly greater ^15^N transfer to barley for both 10 (78.10%) and 20 (82.95%) days compared to WT (68.84% and 77.48% of ^15^N incorporation for 10 and 20 days respectively) (WT v/s Δ*Mep2* (p<0.005)). However, no significant difference in insect derived ^15^N incorporation relative to WT was noted in plants that were not treated with MMN for both 10 and 20 days. Δ*Urease*, showed no significant difference relative to WT in all the experimental condition except 10 days in MMN treated plants. A significantly higher ^15^N incorporation to barley was noted in the presence of Δ*Urease* (79.93%) compared to WT (68.84%) was noted after 10 days in MMN treated plants (WT v/s Δ*Urease* (p<0.0001)).

### Nitrogen source assay

**Fig 6:**
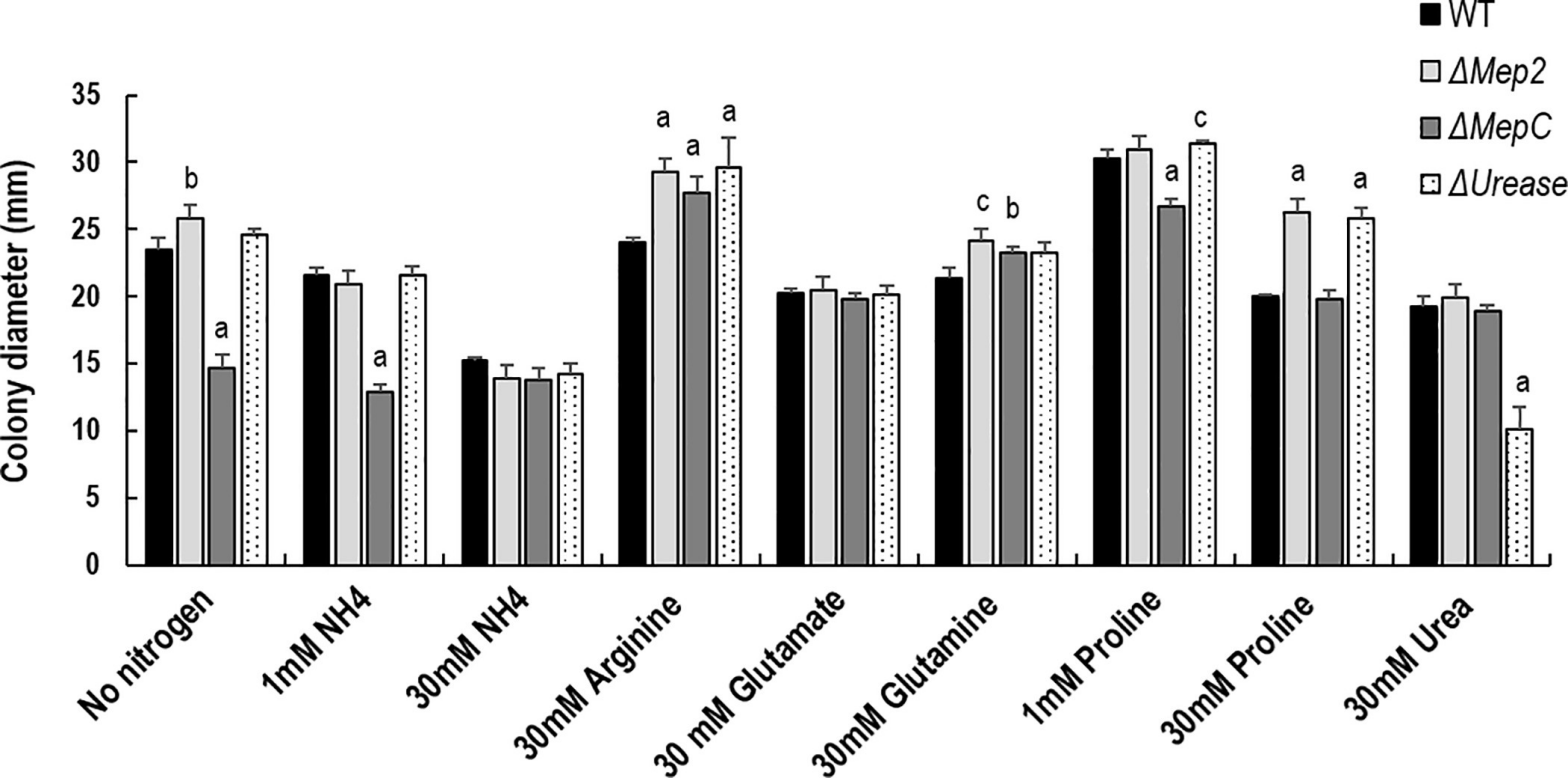
The growth rate of *M*. *robertsii* and mutants on BS medium supplemented with or without different nitrogen sources. The error bars represent the standard error of 5 replicates. The different letters indicate statistically significant differences relative to WT. ‘a’, p <0.001; ‘b’, p<0.01; ‘c’, p<0.04.

The influence of various nitrogen sources on the colony diameter (Fig 6) and colony morphology (S3 Fig) of ammonium permease and *urease* deletions was also assessed. The Δ*MepC* showed reduced colony diameter in the absence or in low concentrations of NH_4_^+^, relative to WT and the colony diameter for Δ*MepC* was similar regardless of the absence of nitrogen (14.66 mm ±0.95), 1 mM NH_4_^+^ (12.91 mm ±0.49) or 30 mM NH_4_^+^ (13.82 mm ±0.84). Compared with WT and other gene deletion strains, Δ*MepC* showed fluffier hyphal growth in low (1 mM) and high (30 mM) NH_4_^+^ (S3 Fig). In contrast, Δ*Mep2* showed a greater colony diameter relative to WT (Tukeys multiple comparison test: p = 0.001) under no nitrogen conditions, while Δ*Urease* exhibited a colony diameter similar to WT in the absence of nitrogen. In 1 mM NH_4_^+^, Δ*Mep2* and Δ*Urease* showed colony diameter similar to the WT, whereas a significantly lower colony diameter relative to WT was noted for the Δ*MepC* (p<0.0001). A significantly greater colony diameter, compared to the WT, was observed for ammonium permease (Δ*MepC* & Δ*Mep2*) and Δ*Urease* when growth in BS agar plates supplemented with either arginine or glutamine (30 mM) as the sole nitrogen source. In 1 mM proline, Δ*MepC* showed reduced colony diameter, while the colony diameter was similar to the WT in 30 mM proline. Interestingly, Δ*Mep2* exhibited a colony diameter similar to the WT in 1 mM proline, whereas a significantly higher colony diameter than WT was noted in 30 mM proline. Δ*Urease* showed less growth in BS agar medium supplemented with urea as a sole nitrogen source (S3 Fig). No significant differences were observed in pH after growing in YPD and transferred to minimal media containing arginine. A slight increase in pH was noted for the WT after 6 hours in glutamine compared to the mutant strains. Additionally, Δ*Mep2* showed an increase in pH relative to WT after 6 hours and 24 hours when transferred to minimal media containing glutamate (S4 Fig).

## Discussion

Here we report the effects of deletions of two ammonium transporters in *M*. *robertsii*, *MepC* and *Mep2* and a sugar transporter *Mrt* on barley root rhizoplane association and the transfer of insect derived nitrogen to plant hosts. The targeted deletion of a *urease* gene in *M*. *robertsii*, however showed no effect on root colonization. The gene deletions, Δ*Pr1A*, Δ*Hypo*. *protein* and Δ*Hyd3*, that were chosen based on the high level of transcripts during root colonization showed no differences in root colonization compared with the WT.

Nitrogen transfer to plants during symbiosis is a common feature described in arbuscular mycorrhizal fungi which is mediated via ammonium permeases [13]. Our study showed that the targeted deletion of *MepC* or *Mep2* in *M*. *robertsii* resulted in enhanced rhizoplane colonization at 10 and 20 days respectively, while the colonization efficiency of Δ*Urease* was unchanged compared to the WT. Ammonium and glutamine are preferred nitrogen sources for fungi. When these nitrogen sources are limited, fungi utilize other nitrogen sources. The regulatory system that enables the selective utilization of secondary nitrogen sources is known as nitrogen metabolite repression or nitrogen catabolite repression [29]. This process allows for the adaptability of fungi to changing nitrogen sources. The expression of AMT/Mep were subjected to nitrogen metabolite repression and this feature was described in non-pathogenic filamentous fungus, *Aspergillus nidulans* [30], phytopathogenic fungi (*Colletotrichum gloeosporioides*) [31] ectomycorrhizal fungi (*Hebeloma cylindrosporum*) [32]. The two GATA transcription factors identified in mediating nitrogen catabolite repression are AreA (*A*. *nidulans*) and nit-2 (*N*. *crassa*) [33]. The functionally equivalent homologues of these regulators known as nitrogen response regulator gene (*nrr1)* are reported in *Metarhizium* and are suggested to play a critical role in insect virulence by regulating subtilisin-like protease and trypsin like protease expression [34]. Similar mechanisms may be operating in *Metarhizium* for plant colonization. The deletion of the ammonium permease gene may have triggered secondary nitrogen utilization pathways which subsequently increased root colonization compared with the WT. The nitrogen source growth assays revealed the differential growth responses of Δ*Mep2* and Δ*MepC* in varying nitrogen conditions. Δ*MepC* showed reduced growth under low nitrogen conditions, while Δ*Mep2* showed no growth impairment. Previous studies demonstrated the differential expression of *mepA* and *mepB* in *A*. *nidulans* which were regulated by the transcription factor AreA [33]. The differential activation of ammonium transporters in response to ammonium and, consequentially, appressoria formation and virulence has been reported in the phytopathogenic fungus *C*. *gloeosporioides*. Their data suggested that the balance between ammonium uptake and release can induce appressorium formation by triggering signaling pathways [31]. Studies have also reported the role of signaling pathways including MAP kinase and cAMP in regulating appressorium formation and virulence in phytopathogenic [35] and entomopathogenic fungi [36,37]. This suggests the role of signaling pathways in sensing host related stimuli (i.e. the form of nitrogen) in initiating fungal colonization on hosts. This phenomenon is not restricted to nitrogen but also to carbon source. For example, *M*. *robertsii* Δ*MrINV* resulted in a severe reduction of growth *in vitro* in root exudate. Conversely it showed enhanced root colonization ability on both switch grass and *Arabidopsis*. The increased root colonization was suggested as catabolite “derepression” in the invertase deletion [8]. Interestingly, no significant differences in endophytic colonization were noted for the nitrogen transporter, Δ*Mrt* or the adhesin gene Δ*Mad2* deletions at 10 and 20 days.

Previous studies has shown that several species of *Metarhizium* including generalist and specialist insect pathogens were able to transfer insect derived nitrogen to plant hosts [4,5]. The ^15^N-transfer assay showed that, the three nitrogen transporter mutants (Δ*MepC*, Δ*Mep2* and Δ*Urease*) showed greater incorporation of insect derived ^15^N to barley plants compared to WT when treated with 50% MMN. This suggests the adaptability of *Metarhizium* to survive in the nitrogen rich environment in the absence of major nitrogen transporters by triggering secondary nitrogen utilization pathways. We observed a significantly low ^15^N incorporation by Δ*MepC* relative to WT in the absence of 50% MMN after 10 days of growth in microcosm. In contrast, the plant colonization assay revealed greater colonization by Δ*MepC* relative to WT after 10 days of association. We speculate that this variation is due to the difference in the nutrient sources available to the fungi in the rhizospheric environment that trigger different compensatory pathways dependent on nutrient availability. Previous studies reported the expression of different subset of genes by *Metarhizium* when colonizing different hosts or in different physiological conditions [15]. Furthermore, studies have shown that the pH in the rhizospheric environment can influence the amount of nitrogen absorbed by the plants [38]. Previous studies demonstrated the significance of pH as an environmental cue in the host niche as a differential gene expression determinant [39].

Fang and St Leger [7], reported that the disruption of the raffinose transporter gene (Δ*Mrt*) greatly reduced the rhizosphere competency of *M*. *robertsii* after 2 and 3 months post inoculation while no significant differences in rhizospheric populations were noted with WT and Δ*Mrt* after 1 week or 1 month [7]. Consistent with this, we found that Δ*Mrt* populations in the rhizosphere remain constant and did not significantly differ from the WT after 10 or 20 days. Surprisingly, we observed enhanced rhizoplane colonization for Δ*Mrt* strains compared to WT at 10 days similar to Δ*MepC*. In contrast, a previous study has shown that Δ*Mrt* was able to colonize corn roots similar to WT but the population in the rhizospheric soil was reduced due to the inability of Δ*Mrt* to uptake oligosaccharides from root exudate [7,40]. The cell wall protein Mad2 is a specific adhesin which enables *M*. *robertsii* to adhere to plant roots. Field trial experiments using corn demonstrated a reduction in survival of Δ*Mad2* in rhizospheric soil as well as reduced ability to colonize plant roots [40]. However, in our study, we did not observe any differences in rhizoplane or rhizospheric association for Δ*Mad2* compared to the WT. This inconsistency in Δ*Mrt* and Δ*Mad2* with regard to root colonization ability could be probably due to the difference in the fungal inoculation method (drench method around planted seedlings) as well as the substrate (vermiculite) we employed for planting, while *Metarhizium* treated corn seeds were used for the field trial study [40].

The targeted deletion of genes (*Pr1A*, *Hyd3* and *Hypo*. *protein*) that were upregulated in plant roots (RNA-Seq data) had no impact on endophytic, rhizoplane or rhizospheric colonization. The deletion of subtilisin-like serine protease (*Pr1A*) did not affect root colonization ability of *M*. *robertsii*. Pr1A is the predominant protease upregulated during *Metarhizium* infection of the insect cuticle [41] and is an important gene used for strain improvement for biocontrol purposes. The integration of multiple copies of *Pr1A* and its constitutive expression in *M*. *anisopliae* increased virulence against insect hosts [42]. The expression of subtilisin-like protease has been reported in plant pathogenic fungi, *Magnaporthe poae*, a pathogen of Kentucky bluegrass. The expression of the protease increased on infected roots [43]. Similar subtilisin-like proteases have also been demonstrated in grass endophytic fungi including *Neotyphodium typhinum* [44] and *Epichloe festucae* [45], however, the specific role of these proteases during plant colonization is unknown. Greater expression levels of *Pr1A* has been demonstrated in *M*. *robertsii* grown in bean root exudate [46], however, evidence suggests that the expression of *Pr1A* is correlated to a stress response and nutrient availability. Hence, the expression of *Pr1A* is proposed as a stress response by *M*. *robertsii* under nutrient starvation conditions including bean root exudate [15] and possibily during plant root colonization. Moreover, previous studies suggested the evolutionary role of different proteases that resulted from gene duplication, loss and horizontal gene transfer events, is correlated with the multiple life styles of *M*. *robertsii* [18].

The role of hydrophobins during initial interaction and virulence with plant hosts have been investigated in phytopathogenic and endophytic fungi [23,47–50]. Although *Hyd3* was upregulated in *Metarhizium-*bean root transcriptome, the disruption of *Hyd3* did not affect the rhizoplane or rhizospheric colonization ability of *M*. *robertsii*. Sequence analysis revealed 96% sequence similarity with *M*. *brunneum HYD3*. However, the loss of the *Hyd3* gene did not affect plant rhizosphere competency for *M*. *brunneum* (unpublished data). Furthermore, previous studies have also revealed the contribution of hydrophobins in the entomopathogenic/endophytic fungus *B*. *bassiana* in root colonization of *Phaseolus vulgaris* [23]. The consequences of the targeted deletion of hydrophobin genes on fungal development and interaction with hosts can vary in different fungi. Besides, the presence or upregulation of other hydrophobins or signaling pathways could compensate or mask homologous gene deletions.

*M*. *robertsii* can infect and kill a wide range of insect species [51]. The molecular and the biochemical factors involved in insect pathogenicity is well studied [2,52]. Disruption of *MepC*, *Mep2*, *urease* or *Hypo*. *protein* gene did not alter the virulence of *M*. *robertsii* against mealworm or wax-moth larvae. However, the disruption of the *Hyd3 or Pr1A* resulted in delayed mortality compared to the WT but only against mealworm larvae. The deletion of *Hyd3*, which encodes a Class I hydrophobin, resulted in altered hydrophobicity and conidiation in *M*. *robertsii*. Consistent with this, the deletion of *HYD3* affected conidiation, surface hydrophobicity and pathogenicity in *M*. *brunneum* [22]. In *B*. *bassiana*, two Class I hydrophobins, hyd1 and hyd2 were reported to play distinct roles in fungal development and interaction with insect hosts. The targeted deletion of *hyd1* significantly altered rodlets on the surface of conidia, lowered surface hydrophobicity and virulence, but the conidia retained adhesion qualities. While the deletion of *hyd2* resulted in decreased surface hydrophobicity and adhesion phenotype but the virulence towards the insect host was not affected [21]. Subtilisin-like serine proteases have been reported in nematode-parasitic, mycoparasitic and entomopathogenic fungi as a virulence factor [53,54]. Subtilisin-like proteases can degrade the protein linkages present on the host integument and thus mediates the penetration and further colonization events during infection. The role of *M*. *robertsii* Pr1A during insect pathogenesis has been reported previously [55].

In conclusion, although high levels of expression of *Pr1A*, *Hypo*. *protein*, *Hyd3* was observed in bean root, the disruption of these genes did not affect the root or rhizosphere colonization ability of the fungus. The deletion of *Hypo*. *protein* had little impact on insect virulence, while *Pr1A* and *Hyd3* contributed to pathogenicity against meal worm. The expression of Hyd3 plays a significant role in conidial hydrophobicity in *M*. *robertsii*. The impact of the gene deletions depends on several features including compensatory gene expression or the plasticity adapted by the organism in order to survive in that environment. The effect of synthetic gene alterations may be effective only under certain environmental or nutrient conditions where the organism is limited in its ability to express compensatory genes [56]. Furthermore, we report the involvement of ammonium permease genes (*MepC* and *Mep2*) of *M*. *robertsii* during plant root colonization as well as in insect derived nitrogen transfer. Nitrogen transporters, *MepC*, *Mep2* and *urease* have no role in insect pathogenicity which was confirmed in two insect hosts. These genes play a critical role in the growth of fungi in nitrogen rich and low conditions. However, a deeper insight into the underlying processes that results the stable association between *M*. *robertsii* and plant host are necessary. The identification and characterization of symbiosis related genes and specific functions will provide a deeper understanding on plant root colonization formed between this ecologically and agronomically important fungus and different plant hosts.

## Supporting information

S1 Table Primer pairs used in the study. (PDF)

S2 Table The top 10 *Metarhizium robertsii* genes that showed increased expression during colonization of *Glycine max* root (RNA-Seq of <1% of total transcripts). (PDF)

S3 Table Endophytic colonization of *M*. *robertsii* WT and mutant strains. (PDF)

S1 Fig **(A) Schematic representation of construction of targeted gene deletion mutants based on homologous recombination and showing a map of a disruption plasmid and its relative position in the *Metarhizium* genome.** The herbicide resistance gene (bar) were inserted in to the open reading frame (ORF) of the target gene. (**B-G) PCR verification of correct integration event in mutants.** (**B)** Confirmation of construction of *MepC* deletion mutant. (**C)** Confirmation of construction of *Mep2* deletion mutant. **(D)** Confirmation of construction of *Urease* deletion mutant. **(E)** Confirmation of construction of *Pr1A* deletion mutant. **(F)** Confirmation of construction of *Hypo*. *protein* deletion mutant. **(G)** Confirmation of construction of *Hyd3* deletion mutant. The top panel of B-G: The PCR conducted with primers bar-up/bar-down and confirmation primer CF2; The PCR products can be obtained only for deletion mutants of each gene not for the wild type (WT). The bottom panel of B-G: The PCR conducted with confirmation primers CF1and CF2. PCR products can be obtained only for WT and not for deletion mutants. M–DNA ladder.
(PDF)

S2 Fig Real-time PCR verification of five *Metarhizium* genes (*Tubulin beta chain* (*Tub*. *beta*), *Hydrophobin* (*Hyd3*), *Hypothetical protein* (*Hypo*. *protein*), *Subtilisin-like serine protease deletion (Pr1A)*, *and Hydrophobin-like protein (ssgA*)) when growing fungus *in vitro* in bean root exudate (BRE) and potato dextrose broth (PDB). The expression of target genes was calibrated by the expression values obtained for the reference gene glyceraldehyde-3-phosphate dehydrogenase (gpd). The relative normalized expression levels were calculated by ΔΔCq method. The error bars represent the standard error of the mean of three biological replicates. ‘a’ indicates statistically significant (p<0.001) difference in expression level of target gene in BRE in comparison to potato dextrose broth PDB.
(PDF)

S3 Fig The colony morphology of WT and mutant strains grown in BS media supplemented with or without different nitrogen sources. (PDF)

S4 Fig The ammonia production based on pH of the mutant strains and WT. 1 ml of the conidial suspension was added on to potato dextrose broth and allowed to grow for 4 days. Mycelia was then filtered, washed with sterile distilled water and transferred to minimal media supplemented with different amino acids. 2.5 g of filtered fungal mycelia was added to 100 ml of minimal media broth and incubated at 100 r.p.m at 27°C. The samples were collected from the cultures was collected at regular intervals (0, 6, 24 and 48 hours) to check the pH. **(A)** Arginine, **(B)** Glutamine, **(C)** Glutamate.
(PDF)

## References

[1] Moonjely S, Barelli L, Bidochka MJ. Insect pathogenic fungi as endophytes In: Lovett B, St. Leger RJ, editors. Insect pathogenic fungi as endophytes. Academic Press Inc.; 2016 p. 107–35.

[2] Ortiz-Urquiza A, Keyhani NO. Action on the surface: Entomopathogenic fungi versus the insect cuticle. Insects. 2013;4(3):357–74. 10.3390/insects4030357 26462424 PMC4553469

[3] Ortiz-Urquiza A, Keyhani NO. Molecular genetics of *Beauveria bassiana* infection of insects In: Lovett B, St. Leger RJ, editors. Molecular genetics of *Beauveria bassiana* infection of insects. Academic Press Inc.; 2016 p. 165–249. 10.1016/bs.adgen.2015.11.003 27131326

[4] Behie SW, Bidochka MJ. Endophytic insect-parasitic fungi translocate nitrogen directly from insects to plants. Science (80). 2012;336:1576–7. 10.1126/science.1222289 22723421

[5] Behie SW, Bidochka MJ. Ubiquity of insect-derived nitrogen transfer to plants by endophytic insect-pathogenic fungi: an additional branch of the soil nitrogen cycle. Appl Environ Microbiol. 2014;80(5):1553–60. 10.1128/AEM.03338-13 24334669 PMC3957595

[6] Behie SW, Moreira CC, Sementchoukova I, Barelli L, Zelisko PM, Bidochka MJ. Carbon translocation from a plant to an insect-pathogenic endophytic fungus. Nat Commun. 2017;8:14245 10.1038/ncomms14245 28098142 PMC5253661

[7] Fang W, St Leger RJ. *Mrt*, a gene unique to fungi, encodes an oligosaccharide transporter and facilitates rhizosphere competency in *Metarhizium robertsii*. Plant Physiol. 2010;154(3):1549–57. 10.1104/pp.110.163014 20837701 PMC2971628

[8] Liao X, Fang W, Lin L, Lu H-L, St Leger RJ. *Metarhizium robertsii* produces an extracellular invertase (MrINV) that plays a pivotal role in rhizospheric interactions and root colonization. PLoS One. 2013;8(10):e78118 10.1371/journal.pone.0078118 24205119 PMC3804458

[9] Ellerbeck M, Schüßler A, Brucker D, Dafinger C, Loos F, Brachmann A. Characterization of three ammonium transporters of the glomeromycotan fungus *Geosiphon pyriformis*. Eukaryot Cell. 2013;12(11):1554–62. 10.1128/EC.00139-13 24058172 PMC3837933

[10] Montanini B, Moretto N, Soragni E, Percudani R, Ottonello S. A high-affinity ammonium transporter from the mycorrhizal ascomycete *Tuber borchii*. Fungal Genet Biol. 2002;36(1):22–34. 10.1016/S1087-1845(02)00001-4 12051892

[11] López-Pedrosa A, González-Guerrero M, Valderas A, Azcón-Aguilar C, Ferrol N. *GintAMT1* encodes a functional high-affinity ammonium transporter that is expressed in the extraradical mycelium of *Glomus intraradices*. Fungal Genet Biol. 2006;43(2):102–10. 10.1016/j.fgb.2005.10.005 16386437

[12] Pérez-Tienda J, Testillano PS, Balestrini R, Fiorilli V, Azcón-Aguilar C, Ferrol N. GintAMT2, a new member of the ammonium transporter family in the arbuscular mycorrhizal fungus *Glomus intraradices*. Fungal Genet Biol. 2011;48(11):1044–55. 10.1016/j.fgb.2011.08.003 21907817

[13] Govindarajulu M, Pfeffer PE, Jin H, Abubaker J, Douds DD, Allen JW, et al Nitrogen transfer in the arbuscular mycorrhizal symbiosis. Nature. 2005;435(7043):819–23. 10.1038/nature03610 15944705

[14] Holsbeeks I, Lagatie O, Van Nuland A, Van De Velde S, Thevelein JM. The eukaryotic plasma membrane as a nutrient-sensing device. Trends Biochem Sci. 2004;29(10):556–64. 10.1016/j.tibs.2004.08.010 15450611

[15] Wang C, Hu G, St. Leger RJ. Differential gene expression by *Metarhizium anisopliae* growing in root exudate and host (*Manduca sexta*) cuticle or hemolymph reveals mechanisms of physiological adaptation. Fungal Genet Biol. 2005;42(8):704–18. 10.1016/j.fgb.2005.04.006 15914043

[16] Freimoser FM, Screen S, Bagga S, Hu G, St Leger RJ. Expressed sequence tag (EST) analysis of two subspecies of *Metarhizium anisopliae* reveals a plethora of secreted proteins with potential activity in insect hosts. Microbiology. 2003;149(1):239–47. 12576597 10.1099/mic.0.25761-0

[17] Hu X, Xiao G, Zheng P, Shang Y, Su Y, Zhang X, et al Trajectory and genomic determinants of fungal-pathogen speciation and host adaptation. Proc Natl Acad Sci. 2014;111(47):16796–801. 10.1073/pnas.1412662111 25368161 PMC4250126

[18] Gao Q, K J, Ying SH, Zhang Y, Xiao G, Shang Y, et al Genome sequencing and comparative transcriptomics of the model entomopathogenic fungi *Metarhizium anisopliae* and *M*. *acridum*. PLoS Genet. 2011;7(1):e1001264 10.1371/journal.pgen.1001264 21253567 PMC3017113

[19] Wang C, St. Leger RJ. The MAD1 adhesin of *Metarhizium anisopliae* links adhesion with blastospore production and virulence to insects, and the MAD2 adhesin enables attachment to plants. Eukaryot Cell. 2007;6(5):808–16. 10.1128/EC.00409-06 17337634 PMC1899246

[20] Xu C, Zhang X, Qian Y, Chen X, Liu R, Zeng G, et al A high-throughput gene disruption methodology for the entomopathogenic fungus *Metarhizium robertsii*. PLoS One. 2014;9(9):e107657 10.1371/journal.pone.0107657 25222118 PMC4164657

[21] Zhang S, Xia YX, Kim B, Keyhani NO. Two hydrophobins are involved in fungal spore coat rodlet layer assembly and each play distinct roles in surface interactions, development and pathogenesis in the entomopathogenic fungus, *Beauveria bassiana*. Mol Microbiol. 2011;80(3):811–26. 10.1111/j.1365-2958.2011.07613.x 21375591

[22] Sevim A, Donzelli BGG, Wu D, Demirbag Z, Gibson DM, Turgeon BG. Hydrophobin genes of the entomopathogenic fungus, *Metarhizium brunneum*, are differentially expressed and corresponding mutants are decreased in virulence. Curr Genet. 2012;58(2):79–92. 10.1007/s00294-012-0366-6 22388867

[23] Moonjely S, Keyhani NO, Bidochka MJ. Hydrophobins contribute to root colonization and stress responses in the rhizosphere-competent insect pathogenic fungus *Beauveria bassiana*. Microbiology. 2018;164(4):517–28. 10.1099/mic.0.000644 29517481

[24] Greenfield M, Gómez-Jiménez MI, Ortiz V, Vega FE, Kramer M, Parsa S. *Beauveria bassiana* and *Metarhizium anisopliae* endophytically colonize cassava roots following soil drench inoculation. Biol Control. 2016;95:40–8. 10.1016/j.biocontrol.2016.01.002 27103778 PMC4825668

[25] Fernandes ÉKK, Keyser CA, Rangel DEN, Foster RN, Roberts DW. CTC medium: A novel dodine-free selective medium for isolating entomopathogenic fungi, especially *Metarhizium acridum*, from soil. Biol Control. 2010;54(3):197–205.

[26] Edgar RC. MUSCLE: multiple sequence alignment with high accuracy and high throughput. Nucleic Acids Res. 2004;32(5):1792–7. 10.1093/nar/gkh340 15034147 PMC390337

[27] Tamura K, Stecher G, Peterson D, Filipski A, Kumar S. MEGA6: Molecular evolutionary genetics analysis version 6.0. Mol Biol Evol. 2013;30(12):2725–9. 10.1093/molbev/mst197 24132122 PMC3840312

[28] Ronquist F, Huelsenbeck JP. MrBayes 3: Bayesian phylogenetic inference under mixed models. Bioinformatics. 2003;19(12):1572–4. 10.1093/bioinformatics/btg180 12912839

[29] Teichert S, Rutherford JC, Wottawa M, Heitman J, Tudzynski B. Impact of ammonium permeases MepA, MepB, and MepC on nitrogen-regulated secondary metabolism in *Fusarium fujikuroi*. Eukaryot Cell. 2008;7(2):187–201. 10.1128/EC.00351-07 18083831 PMC2238153

[30] Monahan BJ, Fraser JA, Hynes MJ, Davis MA. Isolation and characterization of two ammonium permease genes, meaA and mepA, from *Aspergillus nidulans*. Eukaryot Cell. 2002;1(1):85–94. 10.1128/EC.1.1.85-94.2002 12455974 PMC118046

[31] Shnaiderman C, Miyara I, Kobiler I, Sherman A, Prusky D. Differential activation of ammonium transporters during the accumulation of ammonia by *Colletotrichum gloeosporioides* and its effect on appressoria formation and pathogenicity. Mol Plant-Microbe Interact. 2013;26(3):335–55. 10.1094/MPMI-07-12-0170-R 23387470

[32] Javelle A, Morel M, Rodríguez-Pastrana BR, Botton B, André B, Marini AM, et al Molecular characterization, function and regulation of ammonium transporters (Amt) and ammonium-metabolizing enzymes (GS, NADP-GDH) in the ectomycorrhizal fungus *Hebeloma cylindrosporum*. Mol Microbiol. 2003;47(2):411–30. 10.1046/j.1365-2958.2003.03303.x 12519192

[33] Monahan BJ, Askin MC, Hynes MJ, Davis MA. Differential expression of *Aspergillus nidulans* ammonium permease genes is regulated by GATA transcription factor AreA. Eukaryot Cell. 2006;5(2):226–37. 10.1128/EC.5.2.226-237.2006 16467464 PMC1405890

[34] Screen S, Bailey A, Charnley K, Cooper R, Clarkson J. Isolation of a nitrogen response regulator gene (*nrr1*) from *Metarhizium anisopliae*. Gene. 1998;221(1):17–24. 10.1016/s0378-1119(98)00430-2 9852945

[35] Xu JR, Hamer JE. MAP kinase and cAMP signaling regulate infection structure formation and pathogenic growth in the rice blast fungus *Magnaporthe grisea*. Genes Dev. 1996;10(21):2696–706. 10.1101/gad.10.21.2696 8946911

[36] Chen X, Xu C, Qian Y, Liu R, Zhang Q, Zeng G, et al MAPK cascade-mediated regulation of pathogenicity, conidiation and tolerance to abiotic stresses in the entomopathogenic fungus *Metarhizium robertsii*. Environ Microbiol. 2016;18(3):1048–62. 10.1111/1462-2920.13198 26714892

[37] Fang W, Pava-ripoll M, Wang S, St Leger R. Protein kinase A regulates production of virulence determinants by the entomopathogenic fungus, *Metarhizium anisopliae*. Fungal Genet Biol. 2009;46(3):277–85. 10.1016/j.fgb.2008.12.001 19124083

[38] Hawkins BJ, Robbins S. pH affects ammonium, nitrate and proton fluxes in the apical region of conifer and soybean roots. 2010;238–47. 10.1111/j.1399-3054.2009.01317.x 19947965

[39] Zhu J, Ying S-H, Feng M-G. The Pal pathway required for ambient pH adaptation regulates growth, conidiation, and osmotolerance of *Beauveria bassiana* in a pH-dependent manner. Appl Microbiol Biotechnol. 2016;100(10):4423–33. 10.1007/s00253-016-7282-5 26754817

[40] Liao X O’Brien TR, Fang W, St Leger RJ. The plant beneficial effects of *Metarhizium* species correlate with their association with roots. Appl Genet Mol Biotechnol. 2014;98(16):7089–96. 10.1007/s00253-014-5788-2 24805846

[41] Bagga S, Hu G, Screen SE, St Leger RJ. Reconstructing the diversification of subtilisins in the pathogenic fungus *Metarhizium anisopliae*. Gene. 2004;324:159–69. 10.1016/j.gene.2003.09.031 14693381

[42] St Leger RJ, Joshi L, Bidochka MJ, Roberts DW. Construction of an improved mycoinsecticide overexpressing a toxic protease. Proc Natl Acad Sci USA. 1996;93(13):6349–54. 10.1073/pnas.93.13.6349 8692818 PMC39025

[43] Sreedhar L, Kobayashi DY, Bunting TE, Hillman BI, Belanger FC. Fungal proteinase expression in the interaction of the plant pathogen *Magnaporthe poae* with its host. Gene. 1999;235(1):121–9. 10415340 10.1016/s0378-1119(99)00201-2

[44] Reddy P V, Lam CK, Belanger FC. Mutualistic fungal endophytes express a proteinase that is homologous to proteases suspected to be important in fungal pathogenicity. Plant Physiol. 1996;111(4):1209–18. 10.1104/pp.111.4.1209 8756501 PMC160998

[45] Bryant MK, Schardl CL, Hesse U, Scott B. Evolution of a subtilisin-like protease gene family in the grass endophytic fungus *Epichlo festucae*. BMC Evol Biol. 2009;9(1):168. 19615101 10.1186/1471-2148-9-168 PMC2717940

[46] Pava-Ripoll M, Angelini C, Fang W, Wang S, Posada FJ, St Leger R. The rhizosphere-competent entomopathogen *Metarhizium anisopliae* expresses a specific subset of genes in plant root exudate. Microbiology. 2011;157(1):47–55. 20947574 10.1099/mic.0.042200-0

[47] Kim S, Ahn IP, Rho HS, Lee YH. *MHP1*, a *Magnaporthe grisea* hydrophobin gene, is required for fungal development and plant colonization. Mol Microbiol. 2005;57(5):1224–37. 10.1111/j.1365-2958.2005.04750.x 16101997

[48] Viterbo A, Chet I. *TasHyd1*, a new hydrophobin gene from the biocontrol agent *Trichoderma asperellum*, is involved in plant root colonization. Mol Plant Pathol. 2006;7(4):249–58. 10.1111/j.1364-3703.2006.00335.x 20507444

[49] Izumitsu K, Kimura S, Kobayashi H, Morita A, Saitoh Y, Tanaka C. Class I hydrophobin BcHpb1 is important for adhesion but not for later infection of *Botrytis cinerea*. J Gen Plant Pathol. 2010;76(4):254–60.

[50] Dubey MK, Jensen DF, Karlsson M. Hydrophobins are required for conidial hydrophobicity and plant root colonization in the fungal biocontrol agent *Clonostachys rosea*. BMC Microbiol. 2014;14(1):18. 24483277 10.1186/1471-2180-14-18 PMC3922079

[51] Brunner-Mendoza C, del Rocío Reyes-Montes M, Moonjely S, Bidochka MJ, Toriello C. A review on the genus *Metarhizium* as an entomopathogenic microbial biocontrol agent with emphasis on its use and utility in Mexico. Biocontrol Sci Technol. 2018;1–20.

[52] Ortiz-Urquiza A, Keyhani NO. Stress response signaling and virulence: insights from entomopathogenic fungi. Curr Genet. 2015;61(3):239–49. 10.1007/s00294-014-0439-9 25113413

[53] Geremia RA, Goldman GH, Jacobs D, Ardiles W, Vila SB, Van Montagu M, et al Molecular characterization of the proteinase‐encoding gene, *prb1*, related to mycoparasitism by *Trichoderma harzianum*. Mol Microbiol. 1993;8(3):603–13. 10.1111/j.1365-2958.1993.tb01604.x 8326868

[54] Li J, Li Y, Yang J, Dong L, Tian B, Yu Z, et al New insights into the evolution of subtilisin-like serine protease genes in Pezizomycotina. BMC Evol Biol. 2010;10(1):68. 20211028 10.1186/1471-2148-10-68 PMC2848655

[55] Porto M, Leão C, Vieira Tiago P, Dini Andreote F, Luiz De Araújo W, Tinti De Oliveira N. Differential expression of the *pr1A* gene in *Metarhizium anisopliae* and *Metarhizium acridum* across different culture conditions and during pathogenesis. Genet Mol Biol. 2015;38(1):86–92. 10.1590/S1415-475738138120140236 25983629 PMC4415565

[56] Harrison R, Papp B, Pal C, Oliver SG, Delneri D. Plasticity of genetic interactions in metabolic networks of yeast. Proc Natl Acad Sci U S A. 2007;104(7):2307–12. 10.1073/pnas.0607153104 17284612 PMC1892960

