# Supplementary material for: Metarhizium robertsii ammonium permeases (MepC and Mep2) contribute to rhizoplane colonization and modulates the transfer of insect derived nitrogen to plants
Source: PLoS One. 2019 Oct 16;14(10):e0223718. doi: 10.1371/journal.pone.0223718 (PMC6795453; doi:10.1371/journal.pone.0223718)
Supplement: S1 Table — (PDF) [file pone.0223718.s001.pdf]

S1 Table. Primer sequences used in the study.

Primer	Sequence (5'-3')	Function
MAA_04182-5-1	ggTCTAGACCTGACGCACGATGTAG	Disruption of *MAA_04182* (*MepC*)
MAA_04182-5-2	ggGAATTCTAGCAGGCATCGGAGTA	
MAA_04182-3-1	ggACTAGTTGCTTGTTATCCCGTCT	
MAA_04182-3-2	ggCCCGGGCCCTTTGGCTTCTGACT	
MAA_04182-CF1	TCATCGTTTGTGCCATT	Confirmation of the disruption of *MAA_04182* (*MepC*)
MAA_04182-CF2	TGGTTGTCTCCGTGGTG	
MAA_05002-5-1	ggTCTAGATTTCTGGCAGTATGGTC	Disruption of *MAA_05002* (*Mep2*)
MAA_05002-5-2	ggACTAGTATACTTTGCCAAGGTTC	
MAA_05002-3-1	ggACTAGTGACACTGGCAGGGTTGT	
MAA_05002-3-2	ggCCCGGGAGCAGACGGGAGCTTAG	
MAA_05002-CF1	TTCCATTGTATTCAGCC	Confirmation of the disruption of *MAA_05002* (*Mep2*)
MAA_05002-CF2	AATCCTGTTACAGACCG	
MAA_07458-5-1	ggTCTAGACCTCTATCAAGGATGAG	Disruption of *MAA_07458* (Urease)
MAA_07458-5-2	ggGAATTCTATTAGATAGCAACGGA	
MAA_07458-3-1	ggACTAGTAAGAAGGAAACCAAGAA	
MAA_07458-3-2	ggGATATCAGTAAGGAGCGGTGTAT	
MAA_07458-CF1	GCTTCCGAGTGATTTAG	Confirmation of the disruption of *MAA_07458* (Urease)
MAA_07458-CF2	AAGGCTCGCAGAGGTTC	
MAA_05675-5-1	ggACTAGTCGCTAGGCTCTACATTG	Disruption of *MAA_05675* (*Pr1A*)
MAA_05675-5-2	ggACTAGT TGAATCCAGGACAGATC	
MAA_05675-3-1	ggTCTAGATTTCCAACGCTCGAATC	
MAA_05675-3-2	ggCCCGGGGGAACGGGTTGTTTGAG	
MAA_05675-CF1	CAACGGTGCCTAAATTC	Confirmation of the disruption of *MAA_05675* (*Pr1A*)
MAA_05675-CF2	ACGTGTTGGCTAGAATG	
MAA_08959-5-1	ggGCTAGCTCCTTGTGTGATCGTTG	Disruption of *MAA_08959* (*Hypo. protein*)
MAA_08959-5-2	ggGAATTCCGTGTCCAAGTATAGAG	
MAA_08959-3-1	ggGCTAGCCTAGGTCTCGAATACAG	
MAA_08959-3-2	ggGATATCTGCAGCCTTACATTGTC	
MAA_08959-CF1	TGATGAAGACGTGGTTG	Confirmation of the disruption of *MAA_08959* (*Hypo. protein*)
MAA_08959-CF2	ATAGCGGTTCGACCAAG	
MAA_10298-5-1	ggTCTAGACGGATAAGATTCGAGTC	Disruption of *MAA_10298* (*Hyd3*)
MAA_10298-5-2	ggGAATTCTTGTGAGTGAGAGTGAG	
MAA_10298-3-1	ggTCTAGAGCTAGTCAGGTGGTTTG	
MAA_10298-3-2	ggCCCGGGGCTGGTTGGTGTACTTG	

S1 Table – contd.

Primer	Sequence (5'-3')	Function
MAA_10298-CF2<br>MAA_10298-CF2	GCAACCCATGGTTGTAC<br>CTCTTGAGTACGGTAAG	Confirmation of the disruption of *MAA_10298* (*Hyd3*)
Bar-up<br>Bar-down	CGCCTGGACGACTAAACC<br>TCAGCCTGCCGGTACCGC	Confirmation of the disruptions
Tub. beta_F<br>Tub. beta_R	GGCTTCCAGATCACCCACTC<br>GAGAGGGTTGCGTTGTAGGG	Real-time PCR verification
Pr1A_F<br>Pr1A_R	AGTGGCATGGACTACGTTGC<br>GGAGAGGTGTTCTGGGCATC	Real-time PCR verification
Hyd3_F<br>Hyd3_R	CCCATGAGCACAAGAAGCCT<br>GTTCTGGATGGGGAGCAGAG	Real-time PCR verification
Hypo. protein_F<br>Hypo. protein_R	AGCTATACCAGTGGGGACCA<br>AGTAACTTGCGGCGTCTCTT	Real-time PCR verification
gpd_F[Ψ]<br>gpd_R[Ψ]	GTATTGGCCGCATCGTCTTC<br>TCCTTCTTGATGTCGCCCTT	Real-time PCR verification
ssgA_F<br>ssgA_R	GTGTATTGCTGCAACAAAG<br>AGACCATTTTGCTGGACATTG	Real-time PCR verification

* CF2/bar to verify the correct integration of bar gene in target gene. CF1/CF2 to verify the WT and absence of target gene in mutant.

[Ψ] Fang W, Bidochka MJ. Expression of genes involved in germination, conidiogenesis and pathogenesis in *Metarhizium anisopliae* using quantitative real-time RT-PCR. *Mycol Res* 2006; 110:1165–71.
