# Supplementary material for: Metarhizium robertsii ammonium permeases (MepC and Mep2) contribute to rhizoplane colonization and modulates the transfer of insect derived nitrogen to plants
Source: PLoS One. 2019 Oct 16;14(10):e0223718. doi: 10.1371/journal.pone.0223718 (PMC6795453; doi:10.1371/journal.pone.0223718)
Supplement: S2 Table — (PDF) [file pone.0223718.s002.pdf]

S2 Table. The top 10 *Metarhizium robertsii* genes that showed increased expression during colonization of *Glycine max* root (RNA-Seq of <1% of total transcripts).

#	Identification	Gene ID	Number of readings
1	*Putative uncharacterized protein*	*MAA_08959*	234
2	*Hydrophobin*	*MAA_10298*	231
3	*Tubulin beta chain*	*MAA_02081*	155
4	*Putative uncharacterized protein*	*MAA_07571*	131
5	*Subtilisin-like serine protease PR1A*	*MAA_05675*	96
6	*Putative uncharacterized protein*	*MAA_09351*	88
7	*Putative uncharacterized protein*	*MAA_08500*	86
8	*Putative uncharacterized protein*	*MAA_09173*	86
9	*Mmc protein*	*MAA_02991*	77
10	*Hydrophobin-like protein ssgA*	*MAA_09731*	65
