# Supplementary material for: Metarhizium robertsii ammonium permeases (MepC and Mep2) contribute to rhizoplane colonization and modulates the transfer of insect derived nitrogen to plants
Source: PLoS One. 2019 Oct 16;14(10):e0223718. doi: 10.1371/journal.pone.0223718 (PMC6795453; doi:10.1371/journal.pone.0223718)
Supplement: S3 Table — (PDF) [file pone.0223718.s003.pdf]

S3 Table. Endophytic colonization of *M. robertsii* WT and mutant strains.

	# of CFUs/g of root	
**Fungal Strains**	**10 Days**	**20 Days**
WT	424.00 (±358.28)	1419.55 (±936.71)
Δ*MepC*	229.46 (±142.85)	1302.21 (±610.55)
Δ*Mep2*	304.53 (±144.77)	1085.84 (±697.77)
Δ*Urease*	171.80 (±161.50)	1044.53 (±682.97)
Δ*Hyd3*	309.30 (±220.07)	1151.77 (±585.29)
Δ*Pr1A*	194.76 (±126.13)	1062.89 (±736.30)
Δ*Hypo. protein*	152.38 (±121.13)	1040.00 (±664.53)
Δ*Mrt*	352.76 (±149.60)	1478.85 (±594.49)
Δ*Mad2*	310.05 (±115.68)	1087.67 (±281.93)

The standard error of mean (SEM) are indicated in brackets.
