# Supplementary material for: Metarhizium robertsii ammonium permeases (MepC and Mep2) contribute to rhizoplane colonization and modulates the transfer of insect derived nitrogen to plants
Source: PLoS One. 2019 Oct 16;14(10):e0223718. doi: 10.1371/journal.pone.0223718 (PMC6795453; doi:10.1371/journal.pone.0223718)
Supplement: S1 Fig — (A) Schematic representation of construction of targeted gene deletion mutants based on homologous recombination and showing a map of a disruption plasmid and its relative position in the Metarhizium genome. The herbicide resistance gene (bar) were inserted in to the open reading frame (ORF) of the target gene. (B-G) PCR verification of correct integration event in mutants. (B) Confirmation of construction of MepC deletion mutant. (C) Confirmation of construction of Mep2 deletion mutant. (D) Confirmation of construction of Urease deletion mutant. (E) Confirmation of construction of Pr1A deletion mutant. (F) Confirmation of construction of Hypo. protein deletion mutant. (G) Confirmation of construction of Hyd3 deletion mutant. The top panel of B-G: The PCR conducted with primers bar-up/bar-down and confirmation primer CF2; The PCR products can be obtained only for deletion mutants of each gene not for the wild type (WT). The bottom panel of B-G: The PCR conducted with confirmation primers CF1and CF2. PCR products can be obtained only for WT and not for deletion mutants. M–DNA ladder. (PDF) [file pone.0223718.s004.pdf]

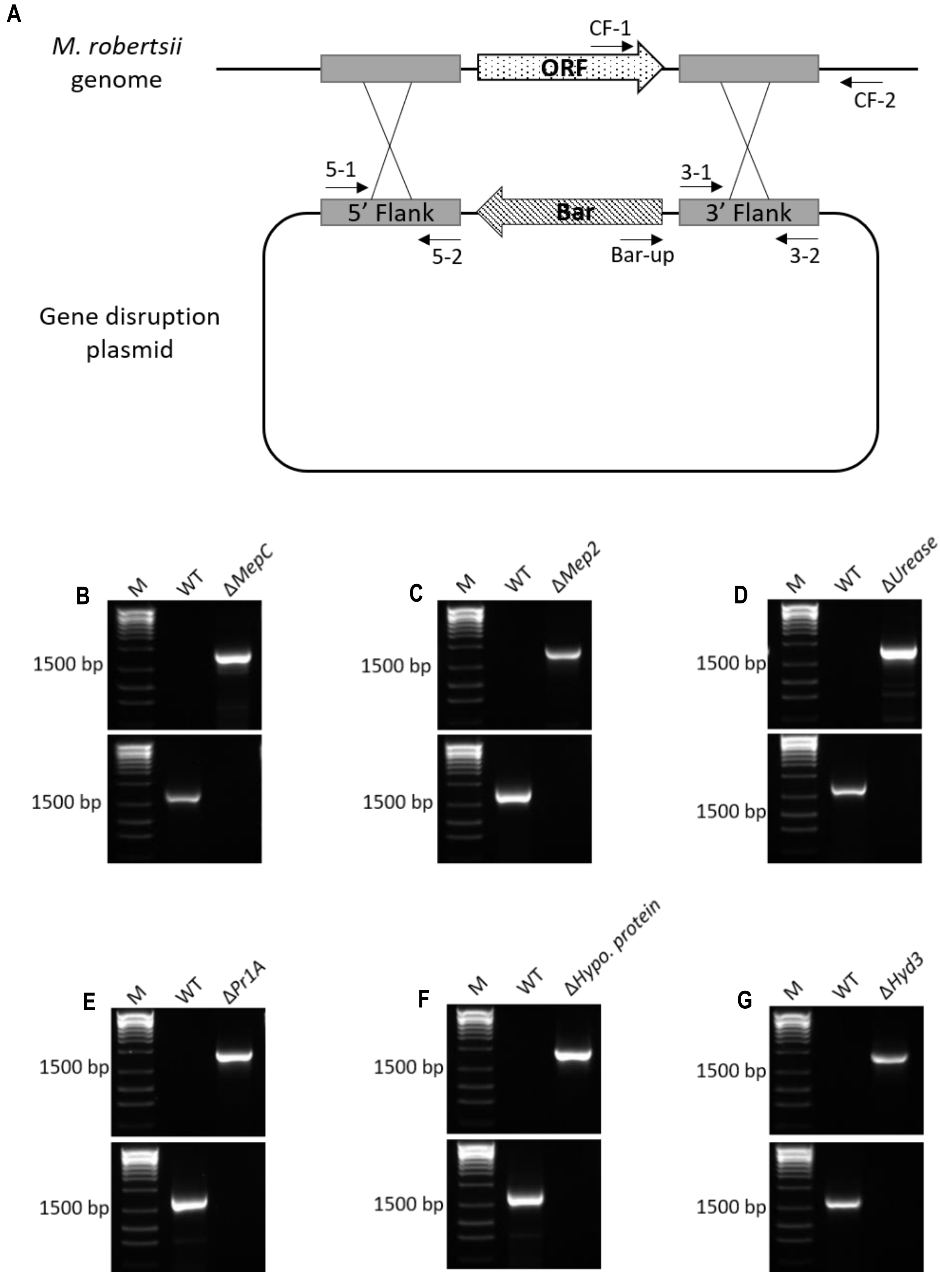

A
M. robertsii genome
CF-1
ORF
CF-2
5-1
3-1
5' Flank
Bar
3' Flank
5-2
Bar-up
3-2
Gene disruption plasmid
B
M
WT
ΔMepC
1500 bp
1500 bp
C
M
WT
ΔMep2
1500 bp
1500 bp
D
M
WT
ΔUrease
1500 bp
1500 bp
E
M
WT
ΔPr1A
1500 bp
1500 bp
F
M
WT
ΔHypo. protein
1500 bp
1500 bp
G
M
WT
ΔHyd3
1500 bp
1500 bp

S1 Fig (A) Schematic representation of construction of targeted gene deletion mutants based on homologous recombination and showing a map of a disruption plasmid and its relative position in the *Metarhizium* genome. The herbicide resistance gene (bar) were inserted in to the open reading frame (ORF) of the target gene. (**B-G) PCR verification of correct integration event in mutants.** (**B)** Confirmation of construction of *MepC* deletion mutant. (**C)** Confirmation of construction of *Mep2* deletion mutant. **(D)** Confirmation of construction of *Urease* deletion mutant. **(E)** Confirmation of construction of *Pr1A* deletion mutant. **(F)** Confirmation of construction of *Hypo. protein* deletion mutant. **(G)** Confirmation of construction of *Hyd3* deletion mutant. The top panel of B-G: The PCR conducted with primers bar-up/bar-down and confirmation primer CF2; The PCR products can be obtained only for deletion mutants of each gene not for the wild type (WT). The bottom panel of B-G: The PCR conducted with confirmation primers CF1and CF2. PCR products can be obtained only for WT and not for deletion mutants. M – DNA ladder.
