# Supplementary material for: Metarhizium robertsii ammonium permeases (MepC and Mep2) contribute to rhizoplane colonization and modulates the transfer of insect derived nitrogen to plants
Source: PLoS One. 2019 Oct 16;14(10):e0223718. doi: 10.1371/journal.pone.0223718 (PMC6795453; doi:10.1371/journal.pone.0223718)
Supplement: S2 Fig — The expression of target genes was calibrated by the expression values obtained for the reference gene glyceraldehyde-3-phosphate dehydrogenase (gpd). The relative normalized expression levels were calculated by ΔΔCq method. The error bars represent the standard error of the mean of three biological replicates. ‘a’ indicates statistically significant (p<0.001) difference in expression level of target gene in BRE in comparison to potato dextrose broth PDB. (PDF) [file pone.0223718.s005.pdf]

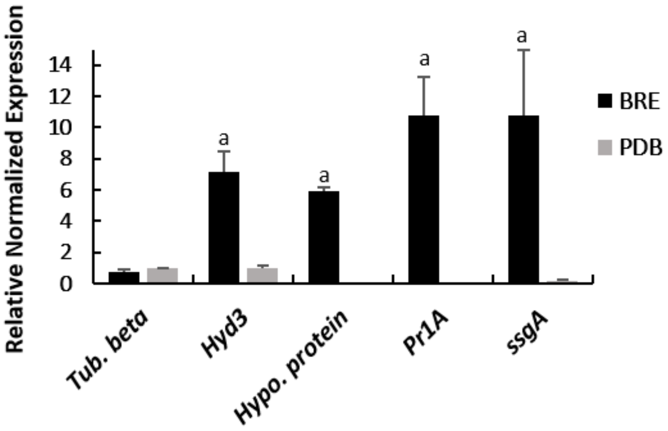

S2 Fig. Real-time PCR verification of five *Metarhizium* genes (*Tubulin beta chain* (*Tub. beta*), *Hydrophobin* (*Hyd3*), *Hypothetical protein* (*Hypo. protein*), *Subtilisin-like serine protease deletion (Pr1A), and Hydrophobin-like protein (ssgA*)) when growing fungus *in vitro* in bean root exudate (BRE) and potato dextrose broth (PDB). The expression of target genes was calibrated by the expression values obtained for the reference gene glyceraldehyde-3-phosphate dehydrogenase (gpd). The relative normalized expression levels were calculated by ΔΔCq method. The error bars represent the standard error of the mean of three biological replicates. ‘a’ indicates statistically significant ($p<0.001$) difference in expression level of target gene in BRE in comparison to potato dextrose broth PDB.
