# Supplementary material for: Metarhizium robertsii ammonium permeases (MepC and Mep2) contribute to rhizoplane colonization and modulates the transfer of insect derived nitrogen to plants
Source: PLoS One. 2019 Oct 16;14(10):e0223718. doi: 10.1371/journal.pone.0223718 (PMC6795453; doi:10.1371/journal.pone.0223718)
Supplement: S3 Fig — (PDF) [file pone.0223718.s006.pdf]

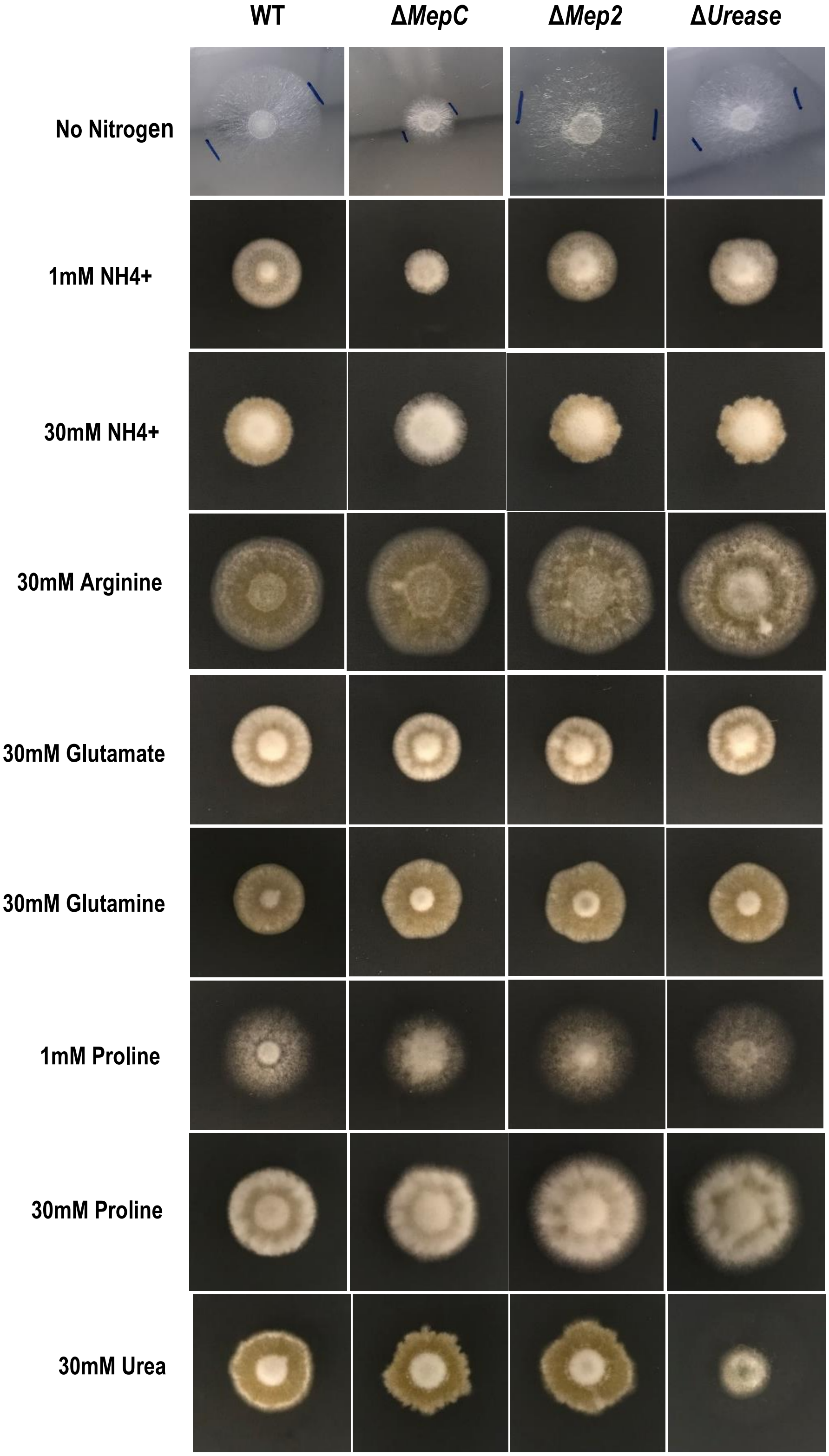

S3 Fig. The colony morphology of WT and mutant strains grown in basal salt media supplemented with or without different nitrogen sources.
