# Supplementary material for: Metarhizium robertsii ammonium permeases (MepC and Mep2) contribute to rhizoplane colonization and modulates the transfer of insect derived nitrogen to plants
Source: PLoS One. 2019 Oct 16;14(10):e0223718. doi: 10.1371/journal.pone.0223718 (PMC6795453; doi:10.1371/journal.pone.0223718)
Supplement: S4 Fig — 1 ml of the conidial suspension was added on to potato dextrose broth and allowed to grow for 4 days. Mycelia was then filtered, washed with sterile distilled water and transferred to minimal media supplemented with different amino acids. 2.5 g of filtered fungal mycelia was added to 100 ml of minimal media broth and incubated at 100 r.p.m at 27°C. The samples were collected from the cultures was collected at regular intervals (0, 6, 24 and 48 hours) to check the pH. (A) Arginine, (B) Glutamine, (C) Glutamate. (PDF) [file pone.0223718.s007.pdf]

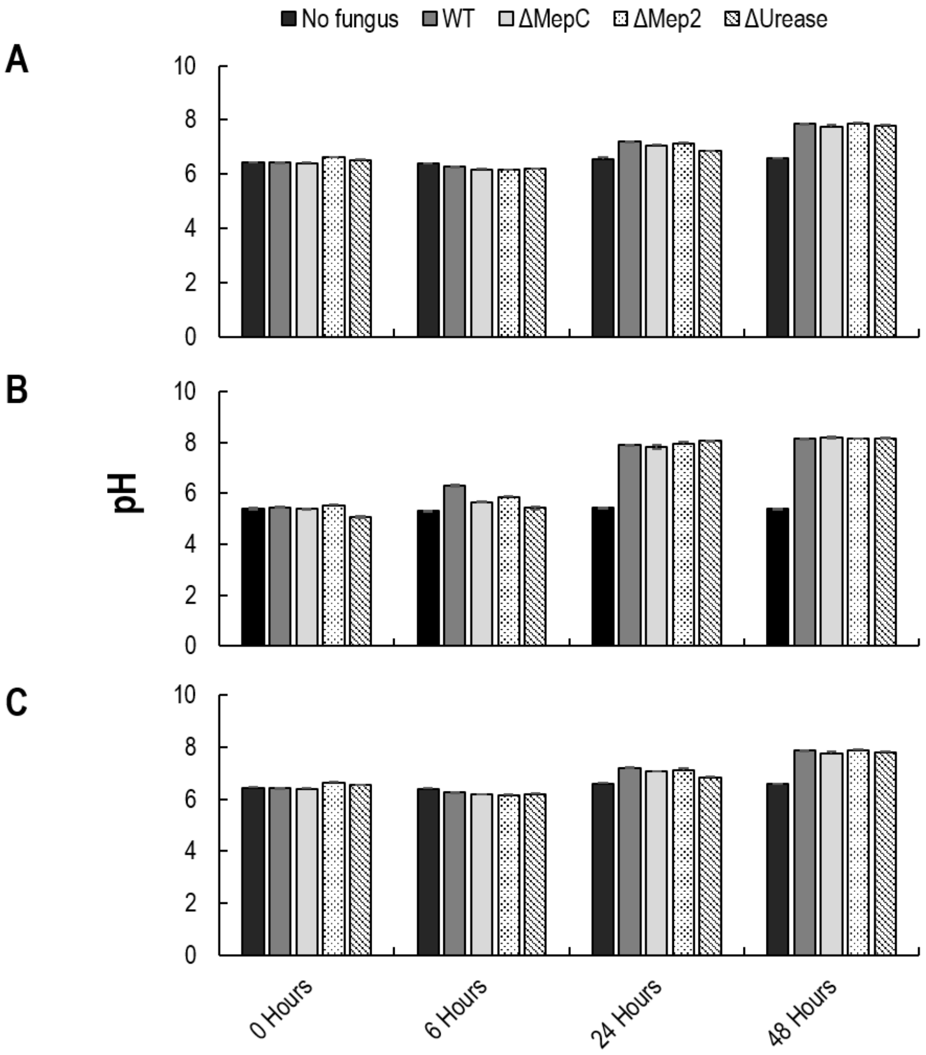

S4 Fig. The ammonia production based on pH of the mutant strains and WT. 1 ml of the conidial suspension was added on to potato dextrose broth and allowed to grow for 4 days. Mycelia was then filtered, washed with sterile distilled water and transferred to minimal media supplemented with different amino acids. 2.5 g of filtered fungal mycelia was added to 100 ml of minimal media broth and incubated at 100 r.p.m at 27°C. The samples were collected from the cultures was collected at regular intervals (0, 6, 24 and 48 hours) to check the pH. **(A)** Arginine, **(B)** Glutamine, **(C)** Glutamate.
